# Supplementary material for: Clustering Accelerometer Activity Patterns from the UK Biobank Cohort
Source: Sensors (Basel). 2021 Dec 9;21(24):8220. doi: 10.3390/s21248220 (PMC8709415; doi:10.3390/s21248220)
Supplement: Supplementary file 1 [file sensors-21-08220-s001.zip › SupplementaryChartsS1toS16_ge100mg.pdf]

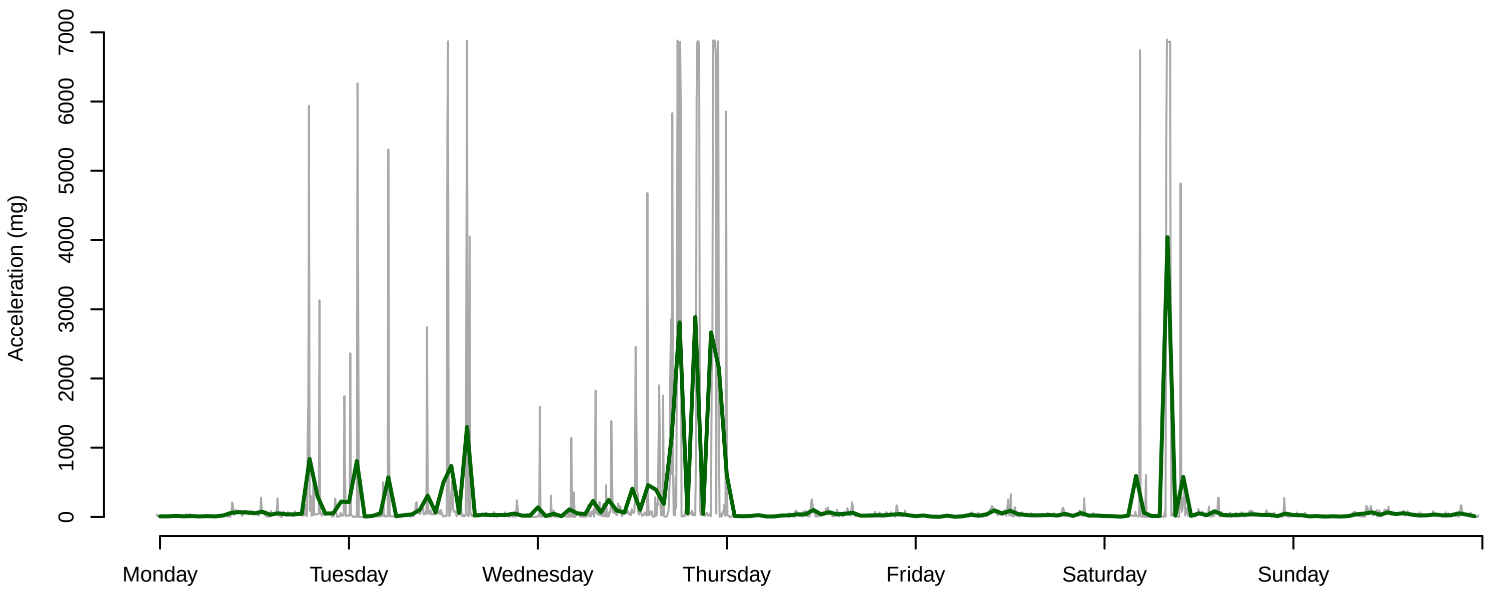

Chart S1 : Participant 1289425

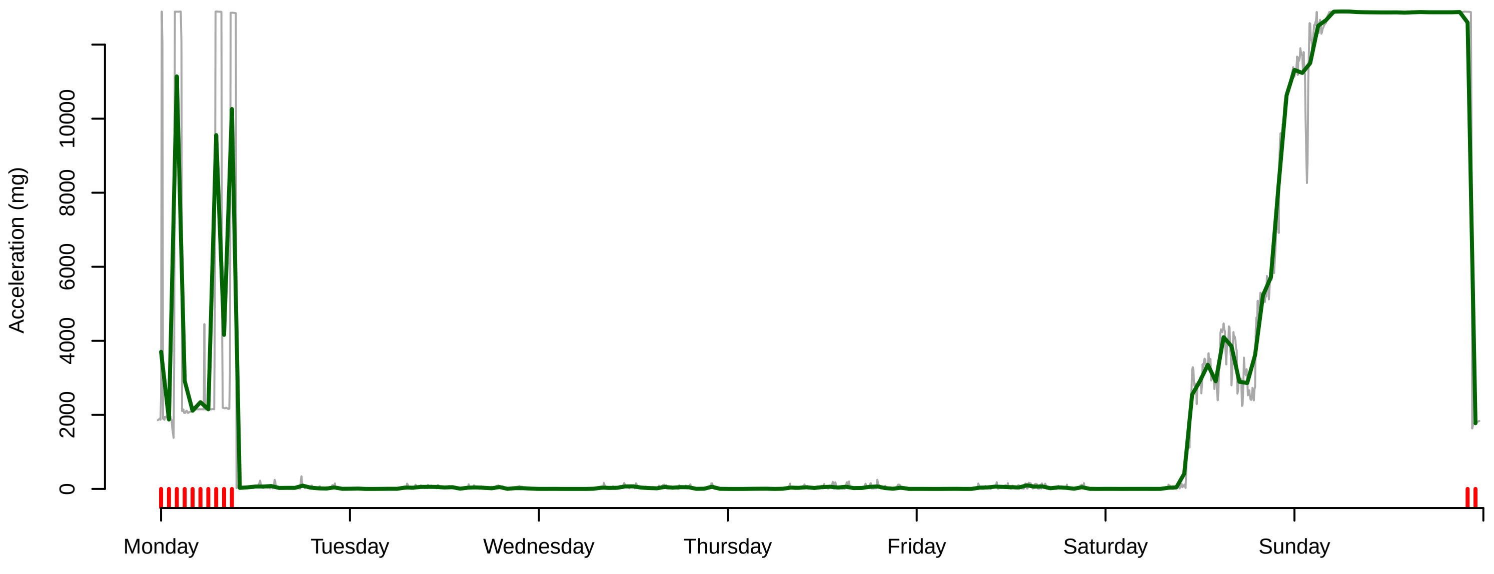

Chart S2 : Participant 1377730

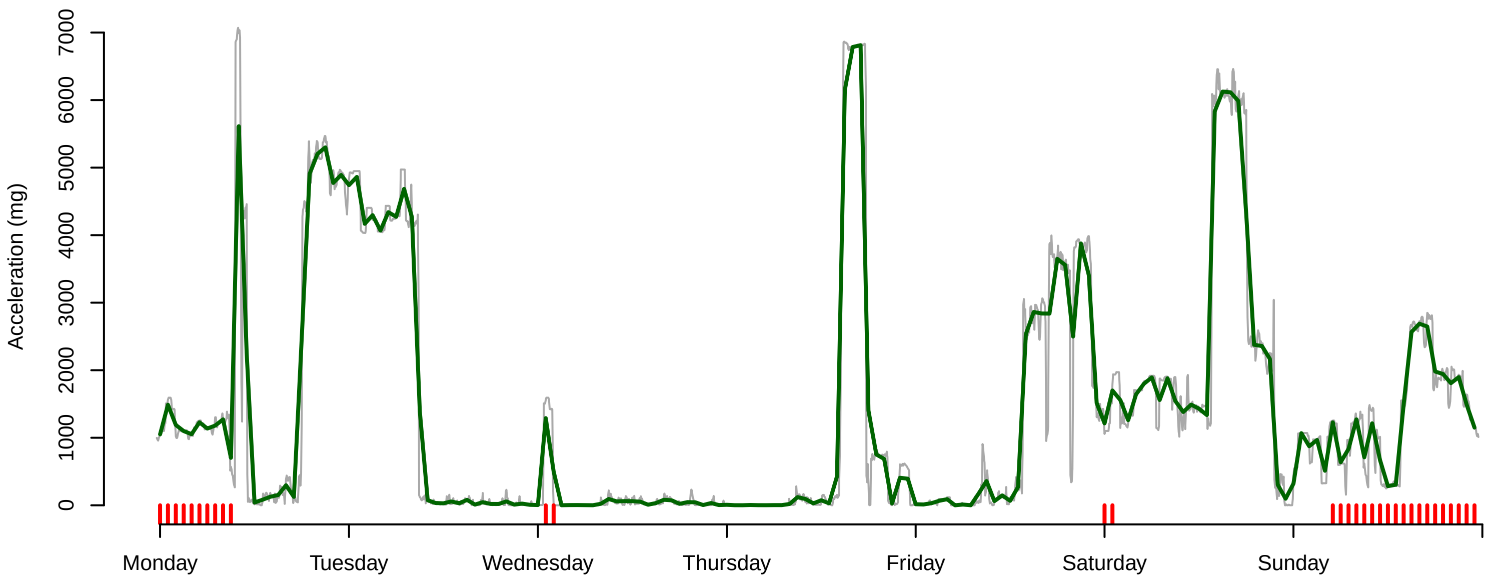

Chart S3 : Participant 1760510

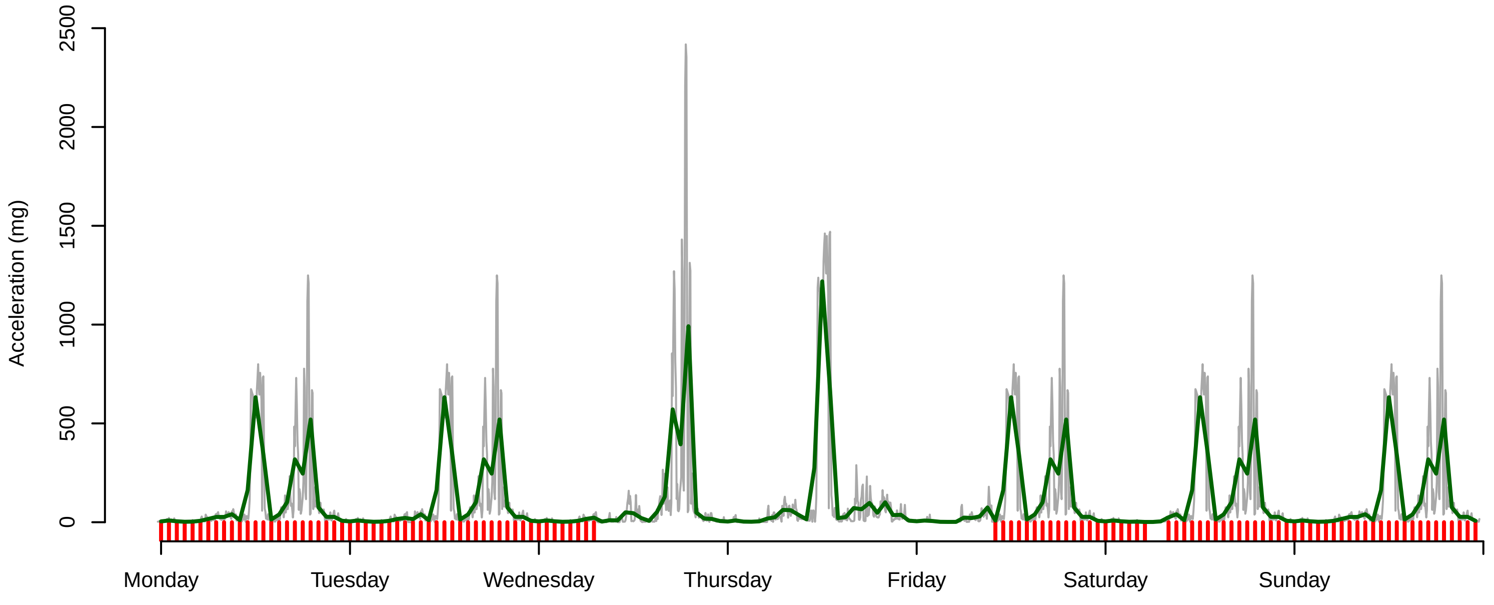

Chart S4 : Participant 1881136

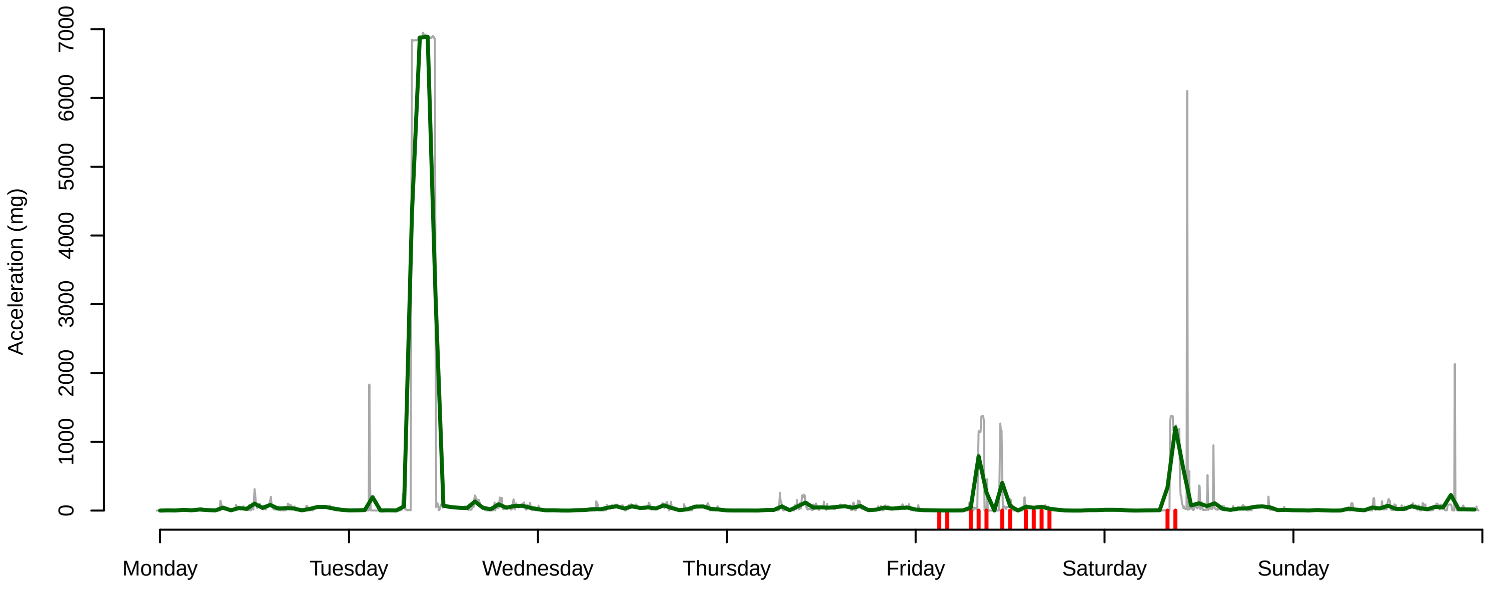

Chart S5 : Participant 3145266

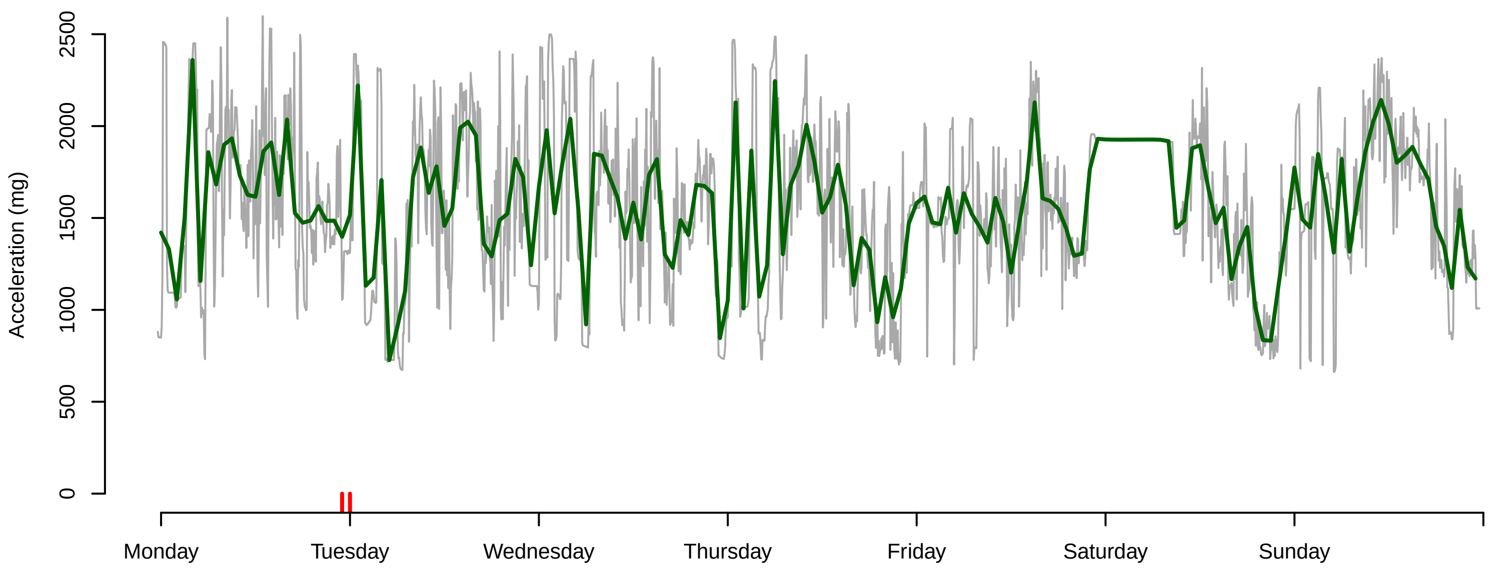

Chart S6 : Participant 3203327

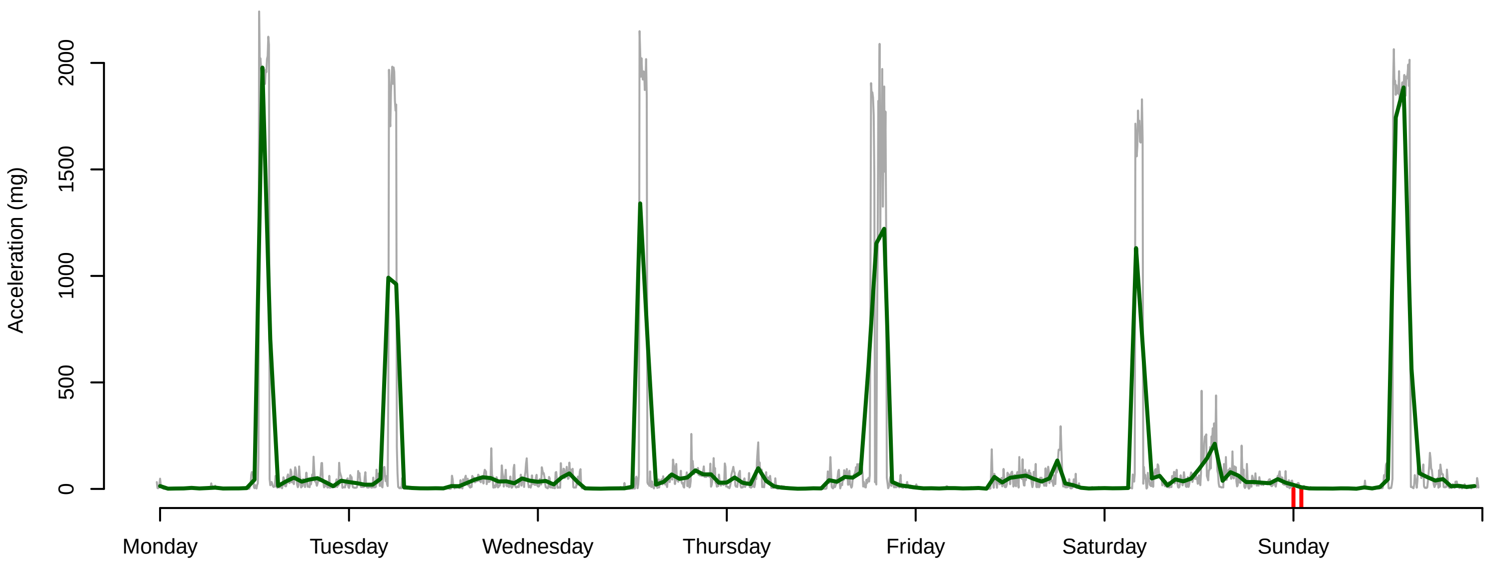

Chart S7 : Participant 3230647

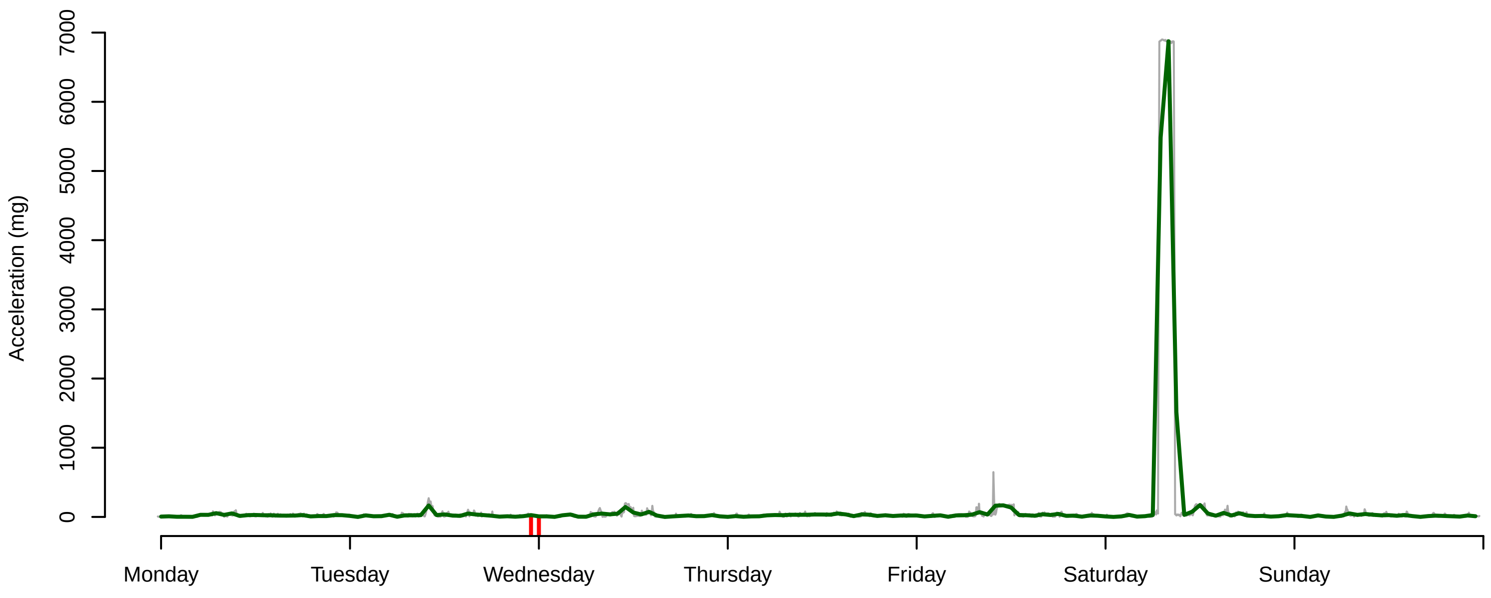

Chart S8 : Participant 3369235

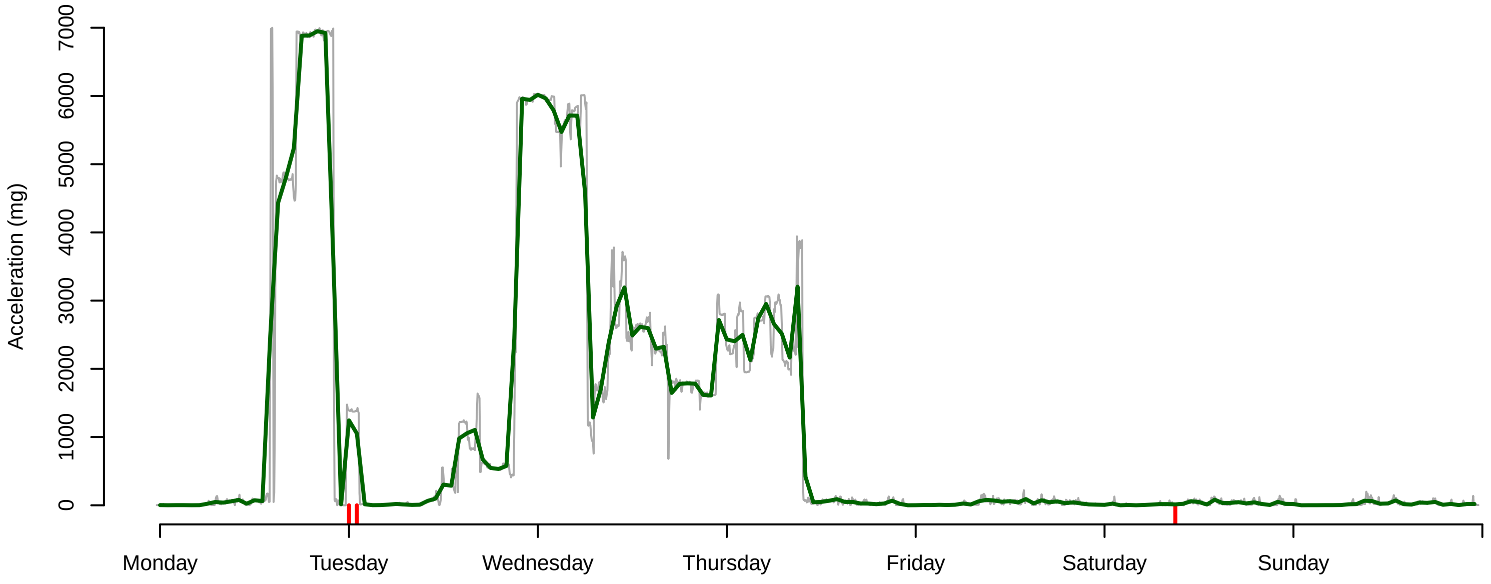

Chart S9 : Participant 3861007

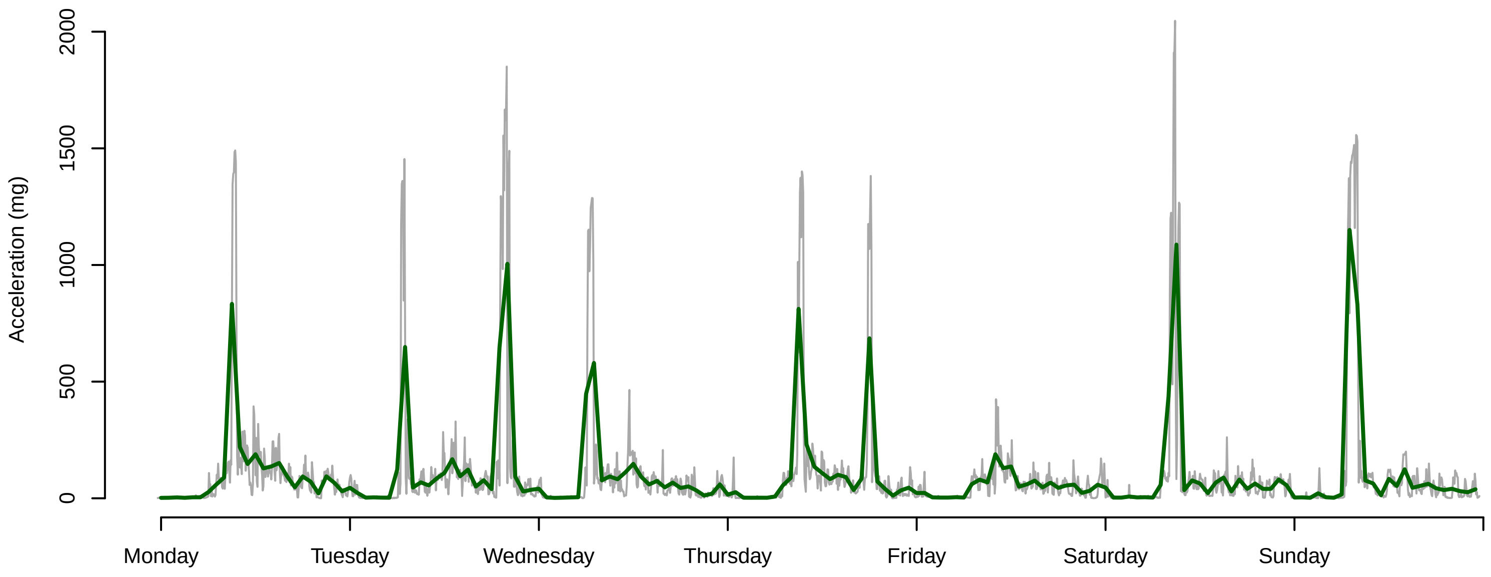

Chart S10 : Participant 3907718

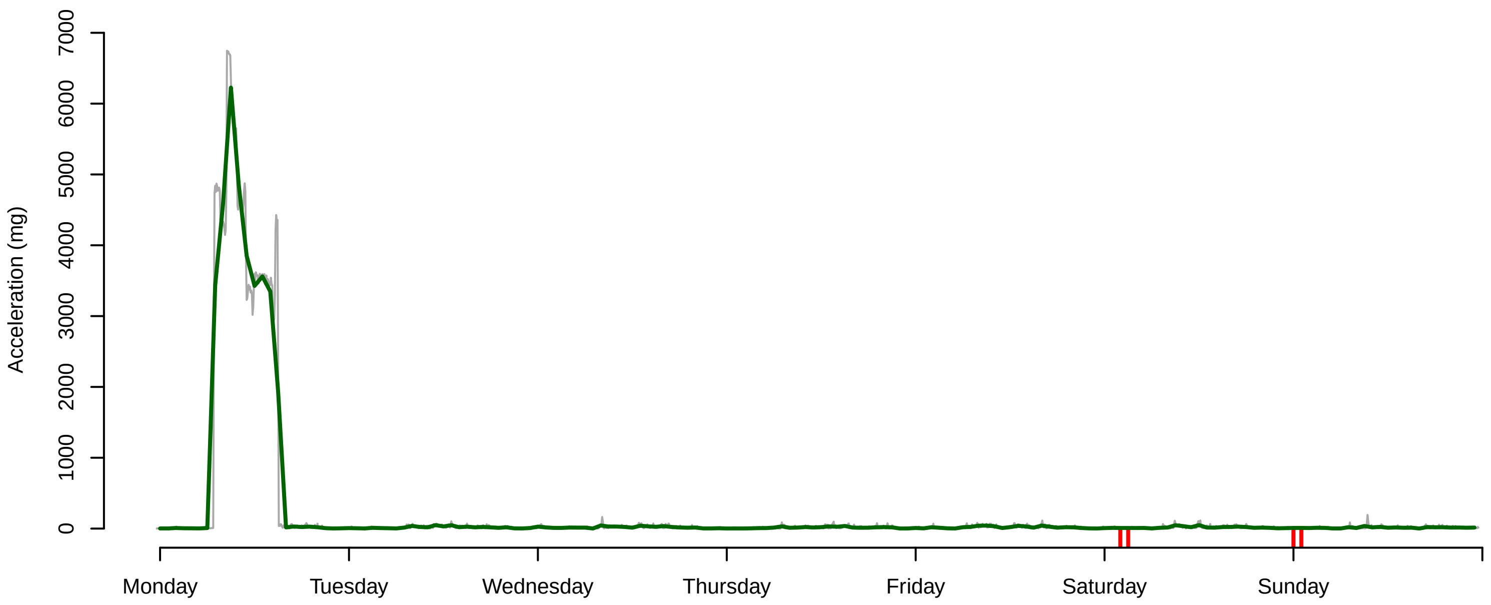

Chart S11 : Participant 4022251

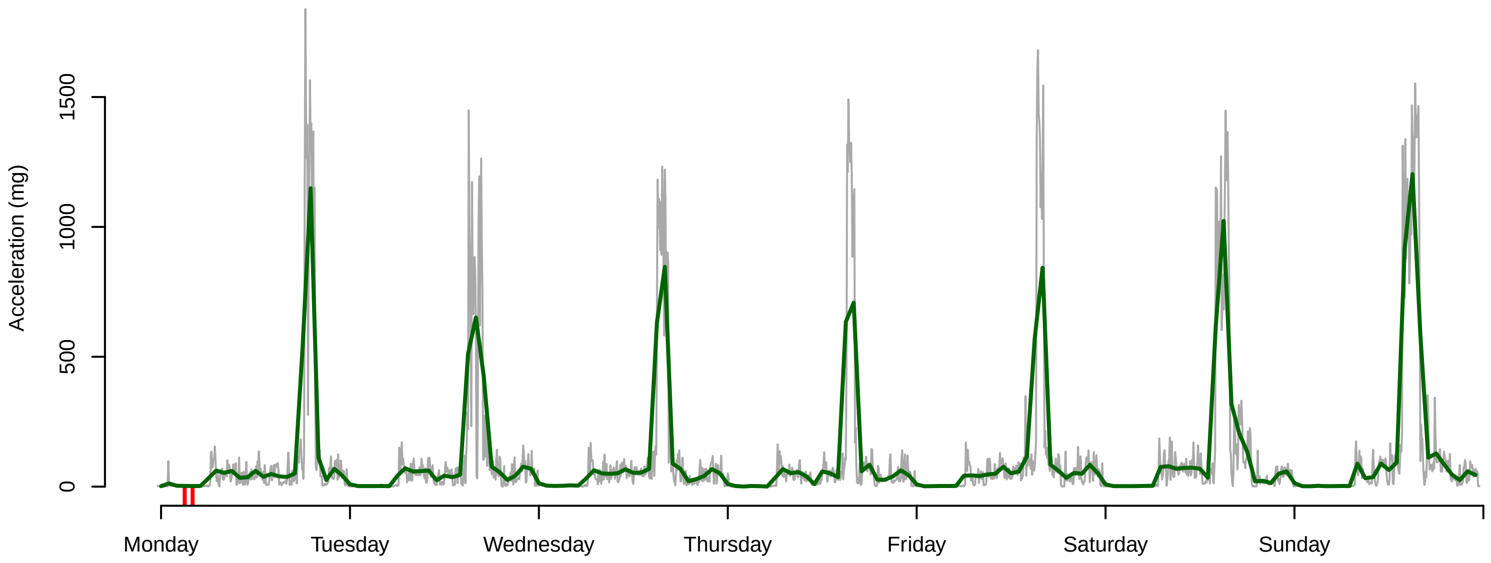

Chart S12 : Participant 4644072

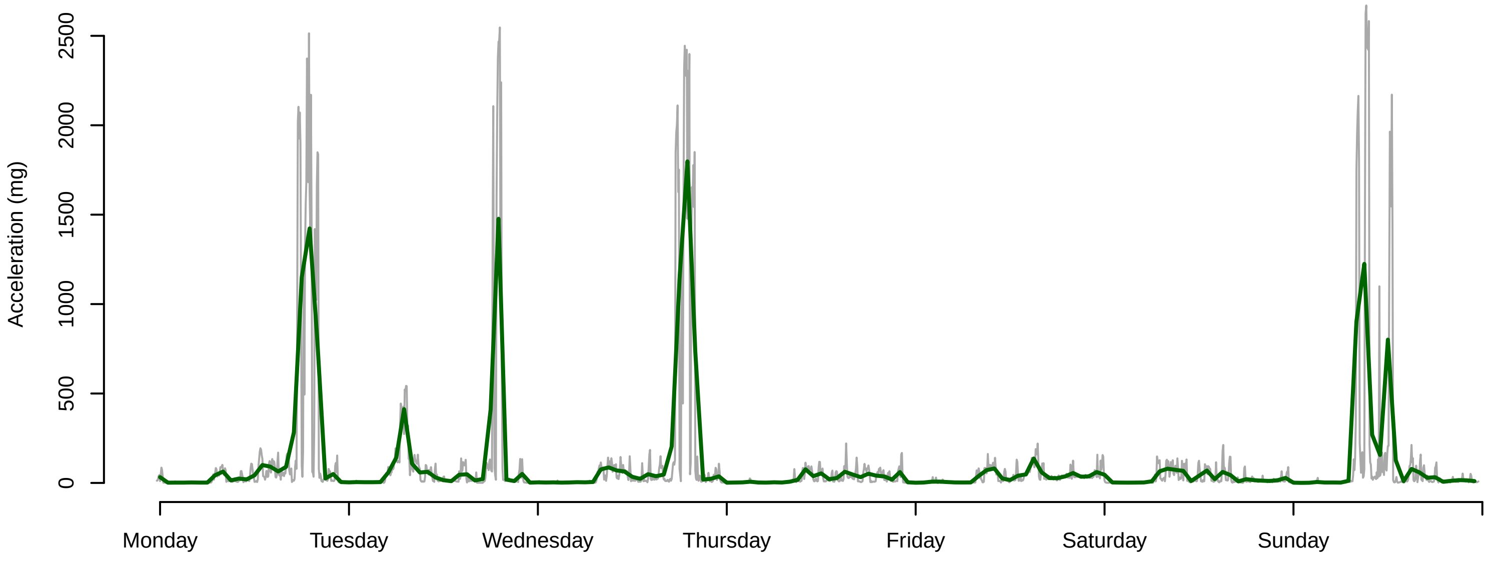

Chart S13 : Participant 4676741

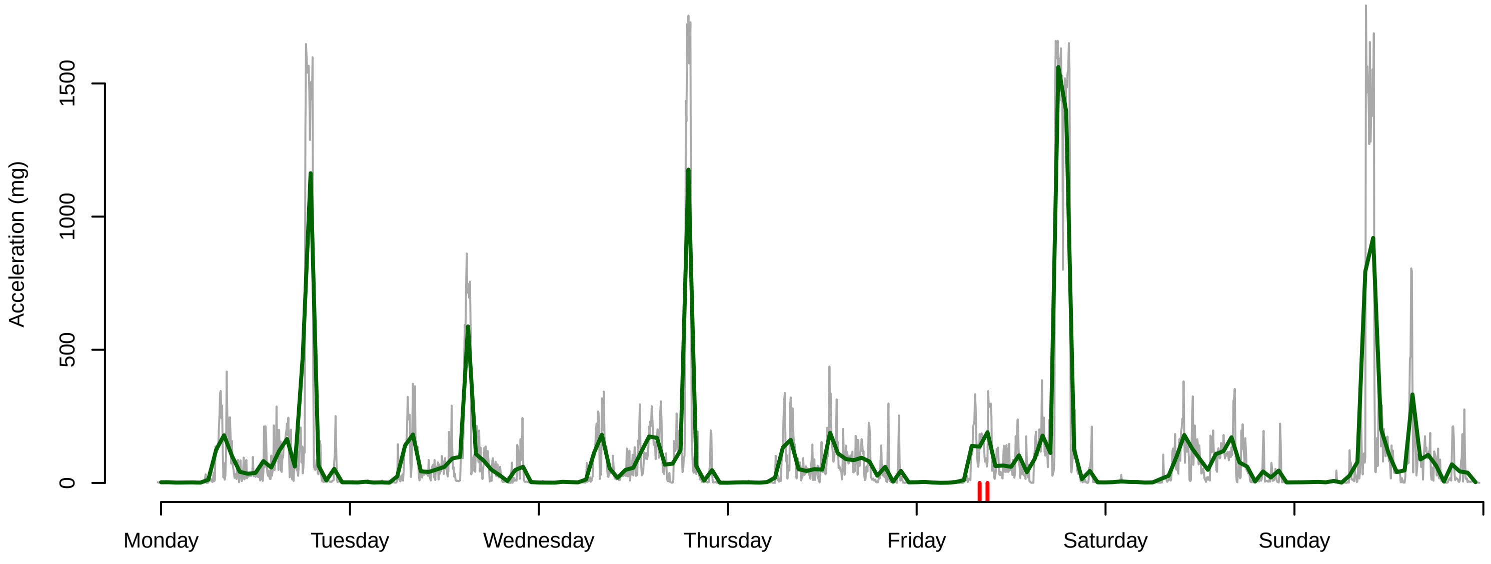

Chart S14 : Participant 5300206

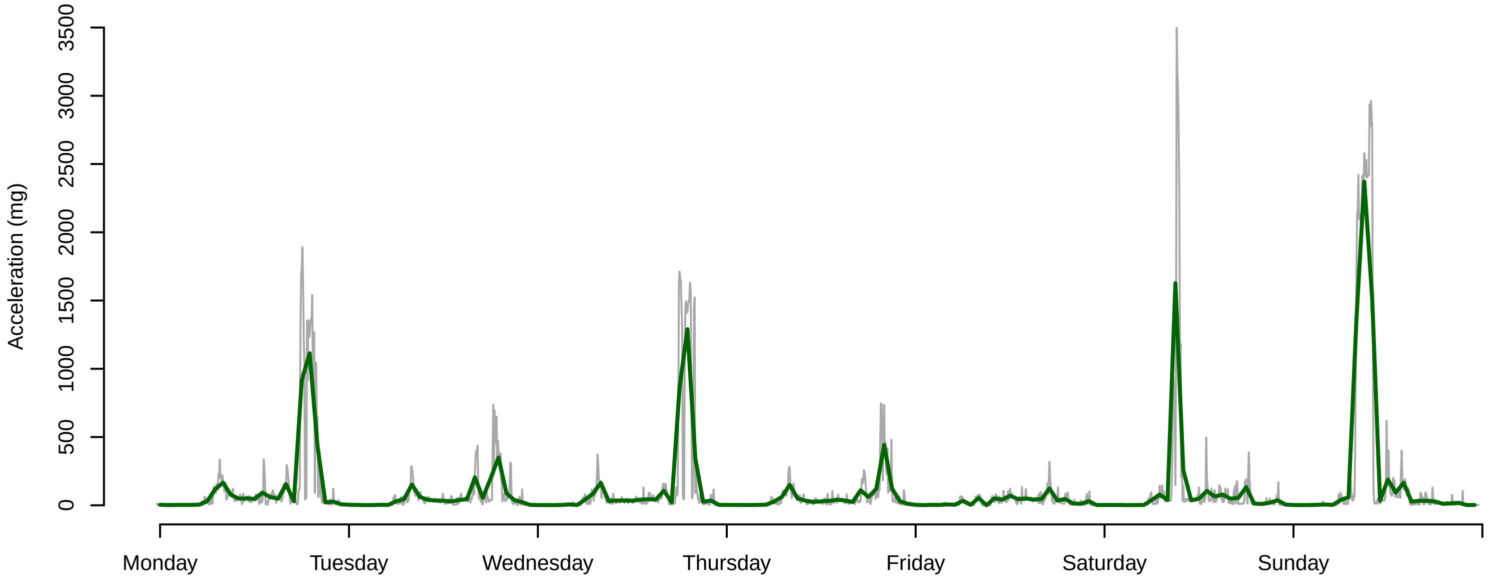

Chart S15 : Participant 5303014

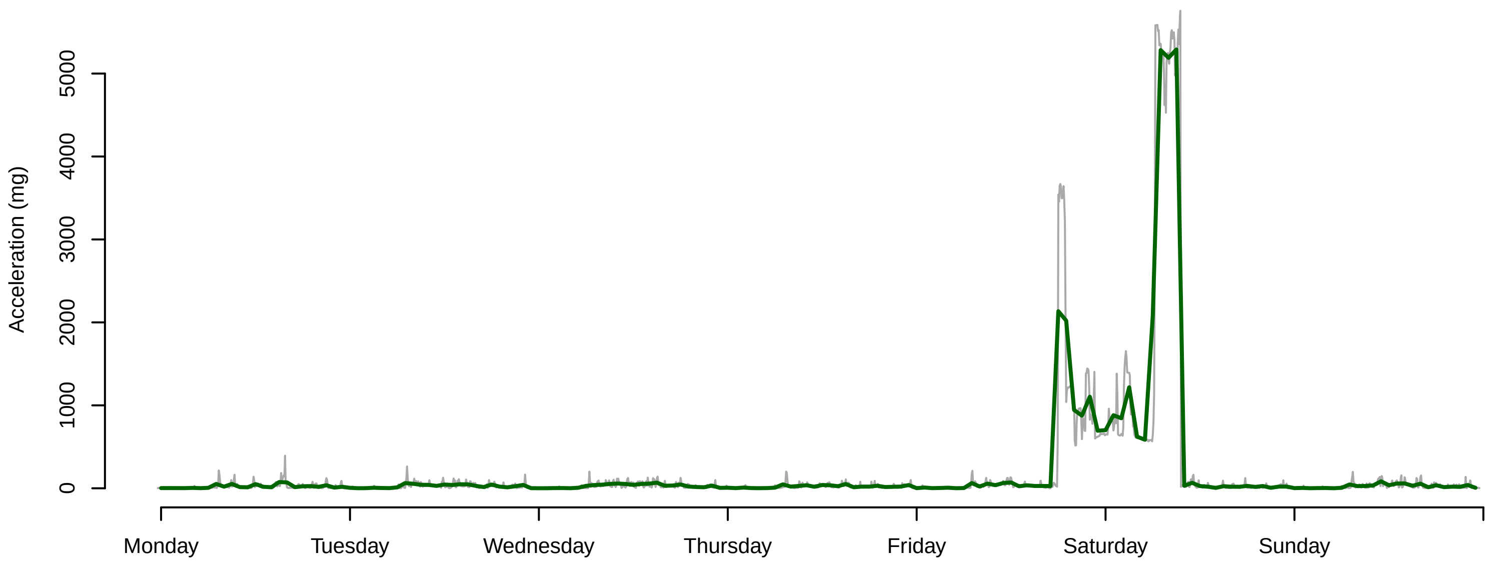

Chart S16 : Participant 5967168
