# Supplementary material for: Clustering Accelerometer Activity Patterns from the UK Biobank Cohort
Source: Sensors (Basel). 2021 Dec 9;21(24):8220. doi: 10.3390/s21248220 (PMC8709415; doi:10.3390/s21248220)
Supplement: Supplementary file 1 [file sensors-21-08220-s001.zip › Supplementaryk_Medoids.pdf]

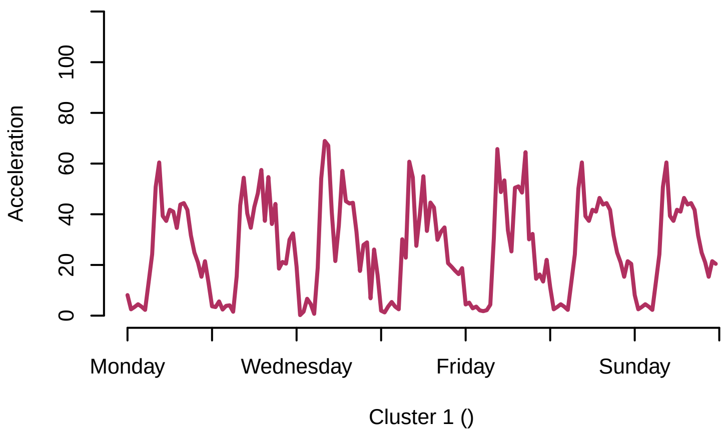
Acceleration
0
20
40
60
80
100
Monday
Wednesday
Friday
Sunday
Cluster 1 ()

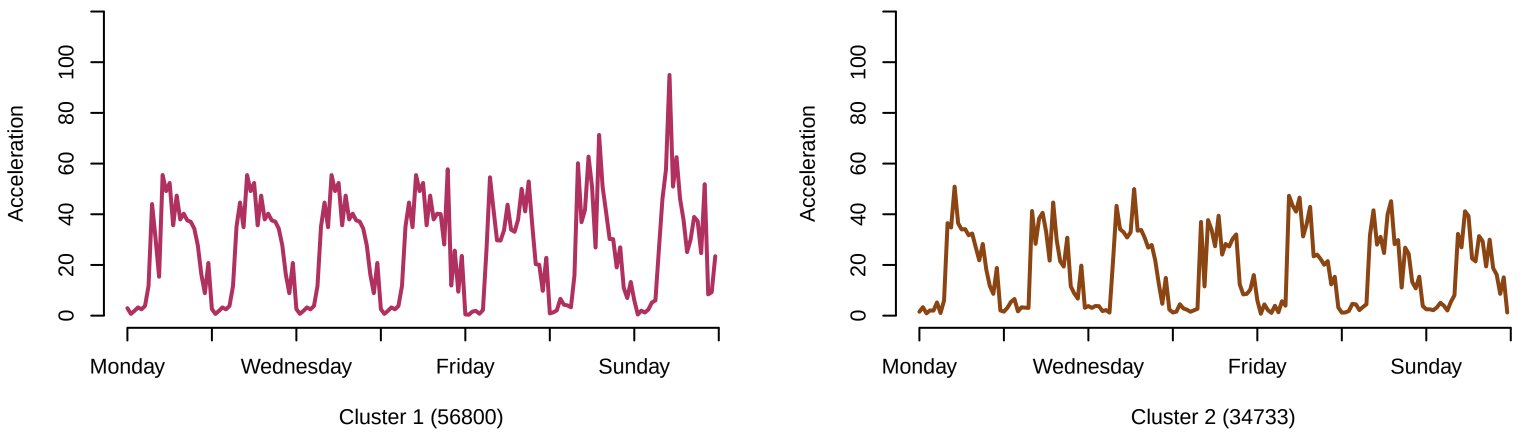
Acceleration
0
20
40
60
80
100
Monday
Wednesday
Friday
Sunday
Cluster 1 (56800)
Acceleration
0
20
40
60
80
100
Monday
Wednesday
Friday
Sunday
Cluster 2 (34733)

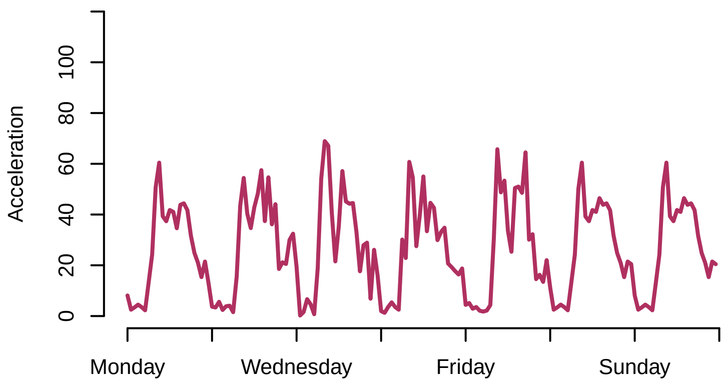

Cluster 1 (42670)

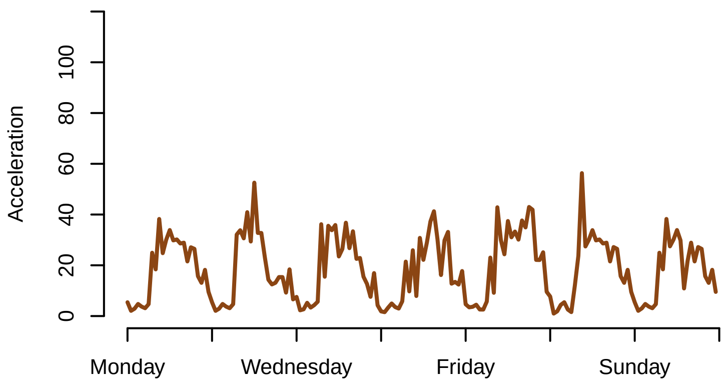

Cluster 2 (32105)

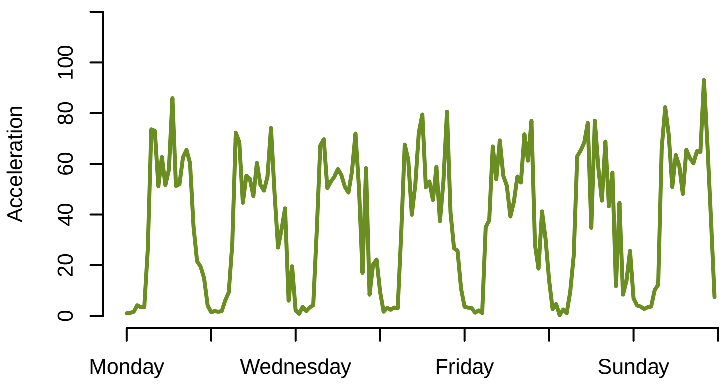

Cluster 3 (16758)

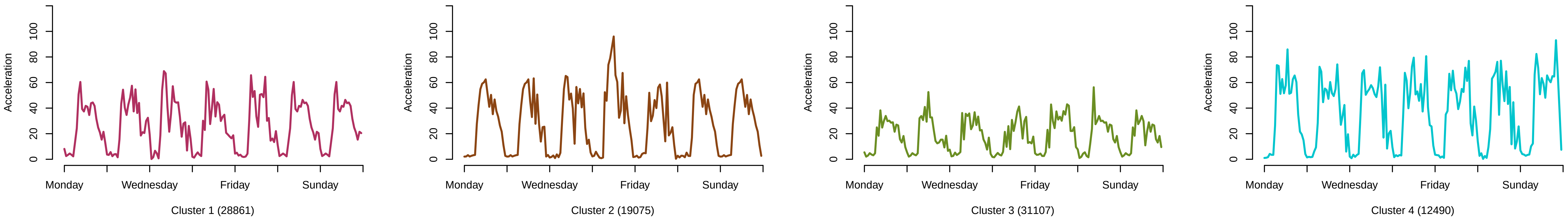
Acceleration
0
20
40
60
80
100
Monday
Wednesday
Friday
Sunday
Cluster 1 (28861)
Cluster 2 (19075)
Cluster 3 (31107)
Cluster 4 (12490)

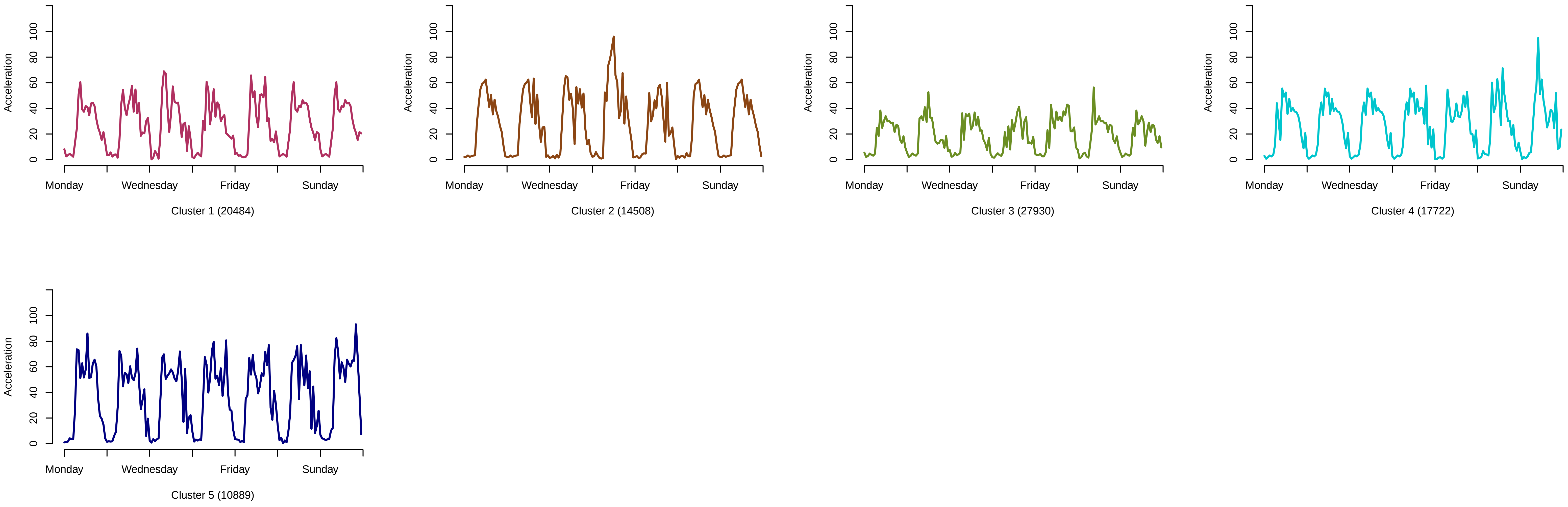
Acceleration
0
20
40
60
80
100
Monday
Wednesday
Friday
Sunday
Cluster 1 (20484)
Cluster 2 (14508)
Cluster 3 (27930)
Cluster 4 (17722)
Cluster 5 (10889)

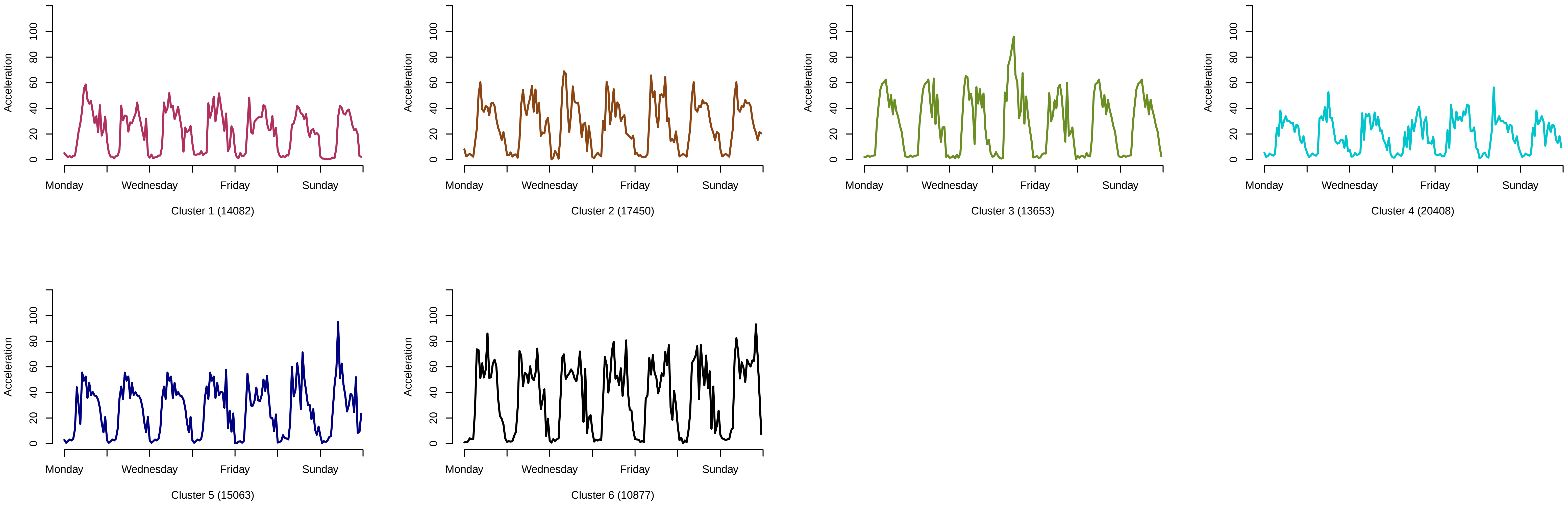
Acceleration
0
20
40
60
80
100
Monday
Wednesday
Friday
Sunday
Cluster 1 (14082)
Cluster 2 (17450)
Cluster 3 (13653)
Cluster 4 (20408)
Cluster 5 (15063)
Cluster 6 (10877)

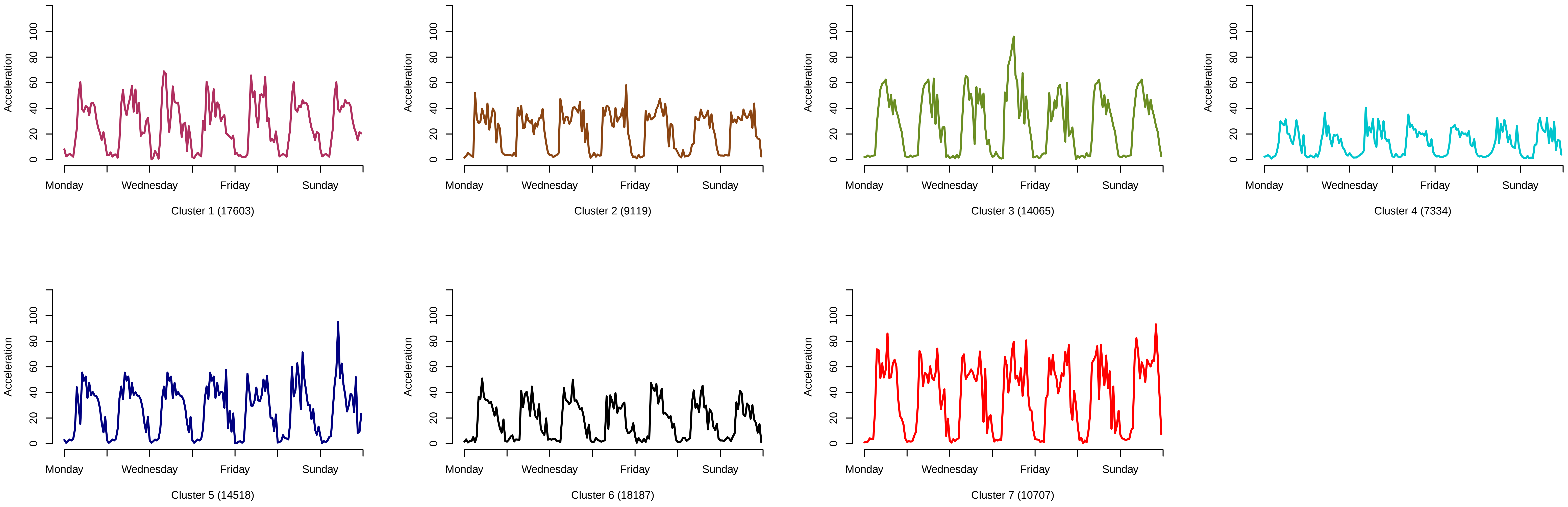
Acceleration
0
20
40
60
80
100
Monday
Wednesday
Friday
Sunday
Cluster 1 (17603)
Cluster 2 (9119)
Cluster 3 (14065)
Cluster 4 (7334)
Cluster 5 (14518)
Cluster 6 (18187)
Cluster 7 (10707)

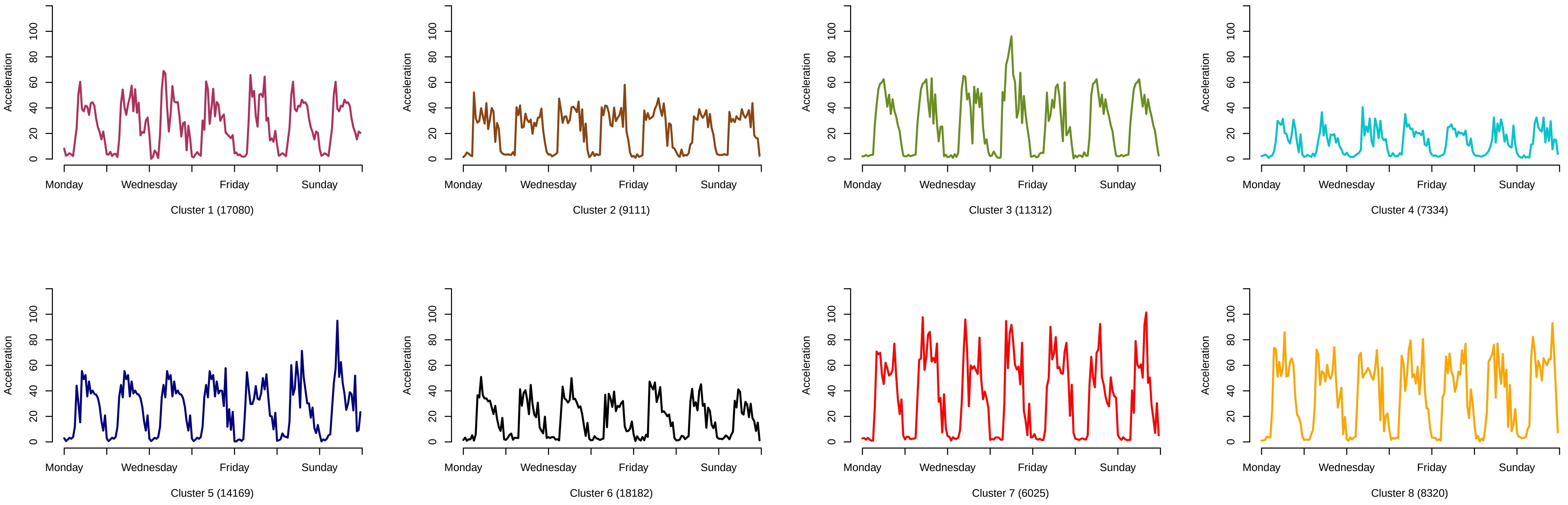
Acceleration
0
20
40
60
80
100
Monday
Wednesday
Friday
Sunday
Cluster 1 (17080)
Cluster 2 (9111)
Cluster 3 (11312)
Cluster 4 (7334)
Cluster 5 (14169)
Cluster 6 (18182)
Cluster 7 (6025)
Cluster 8 (8320)

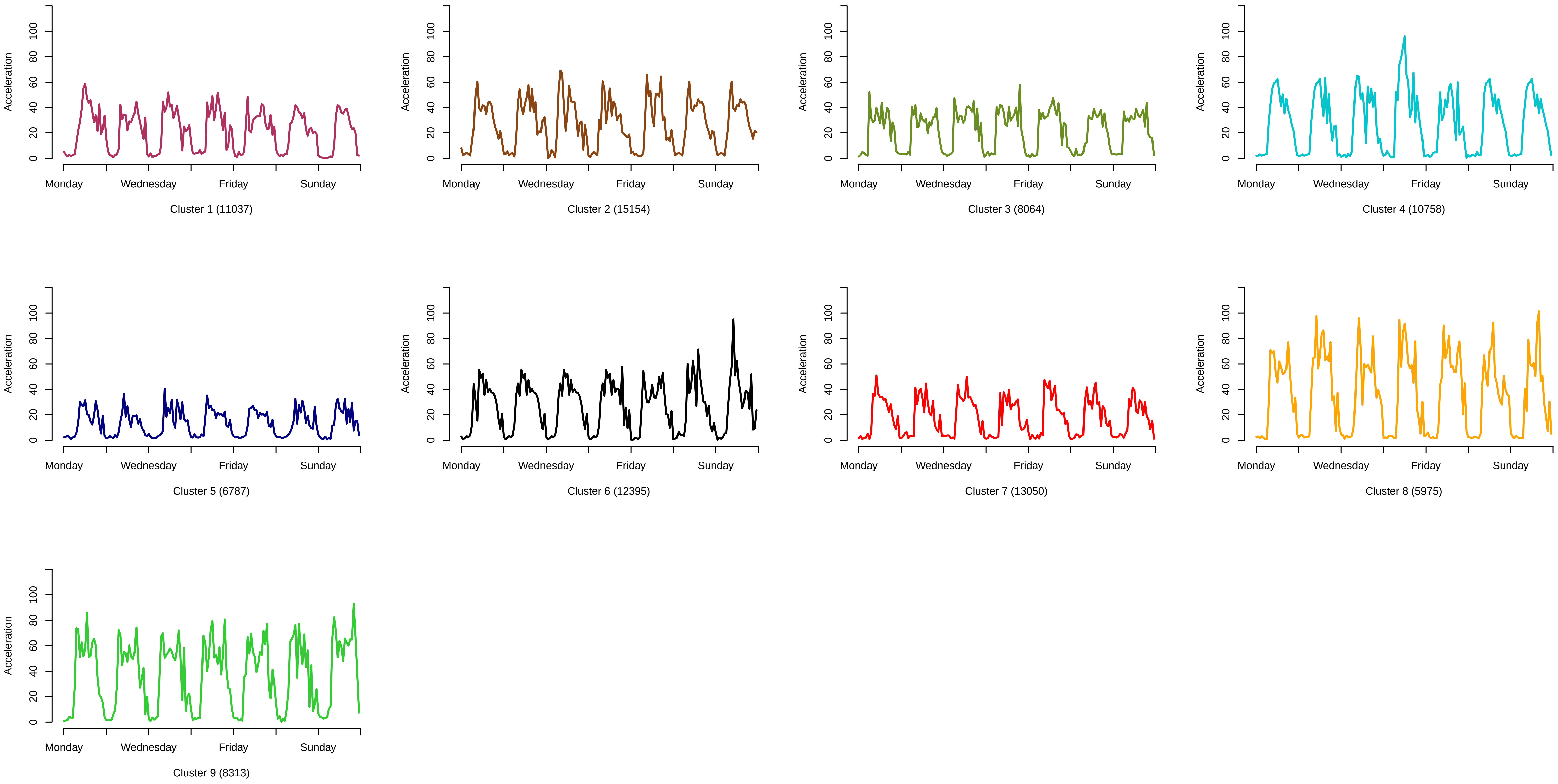
Acceleration
0
20
40
60
80
100
Monday
Wednesday
Friday
Sunday
Cluster 1 (11037)
Cluster 2 (15154)
Cluster 3 (8064)
Cluster 4 (10758)
Cluster 5 (6787)
Cluster 6 (12395)
Cluster 7 (13050)
Cluster 8 (5975)
Cluster 9 (8313)

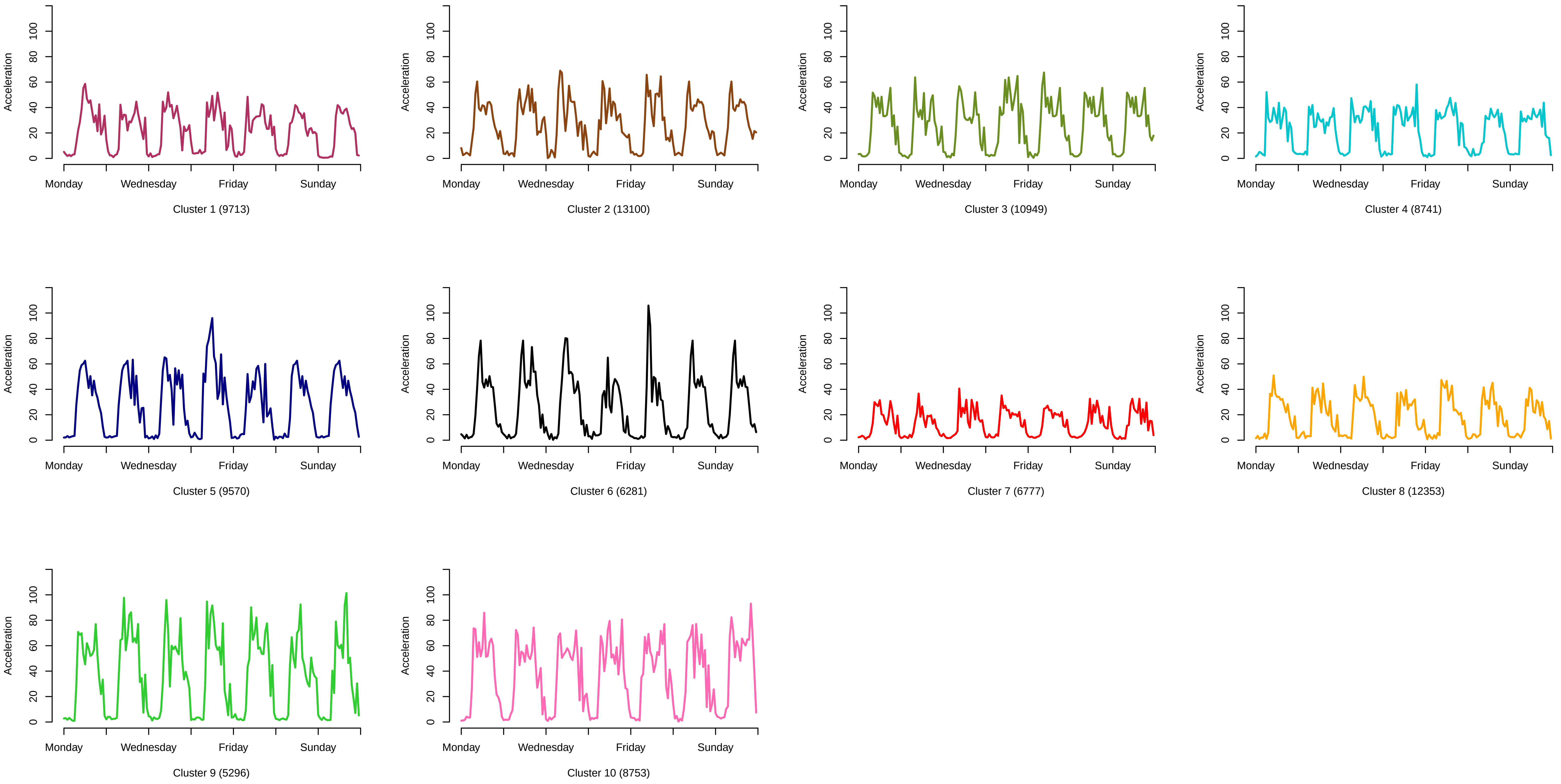
Acceleration
0
20
40
60
80
100
Monday
Wednesday
Friday
Sunday
Cluster 1 (9713)
Cluster 2 (13100)
Cluster 3 (10949)
Cluster 4 (8741)
Cluster 5 (9570)
Cluster 6 (6281)
Cluster 7 (6777)
Cluster 8 (12353)
Cluster 9 (5296)
Cluster 10 (8753)

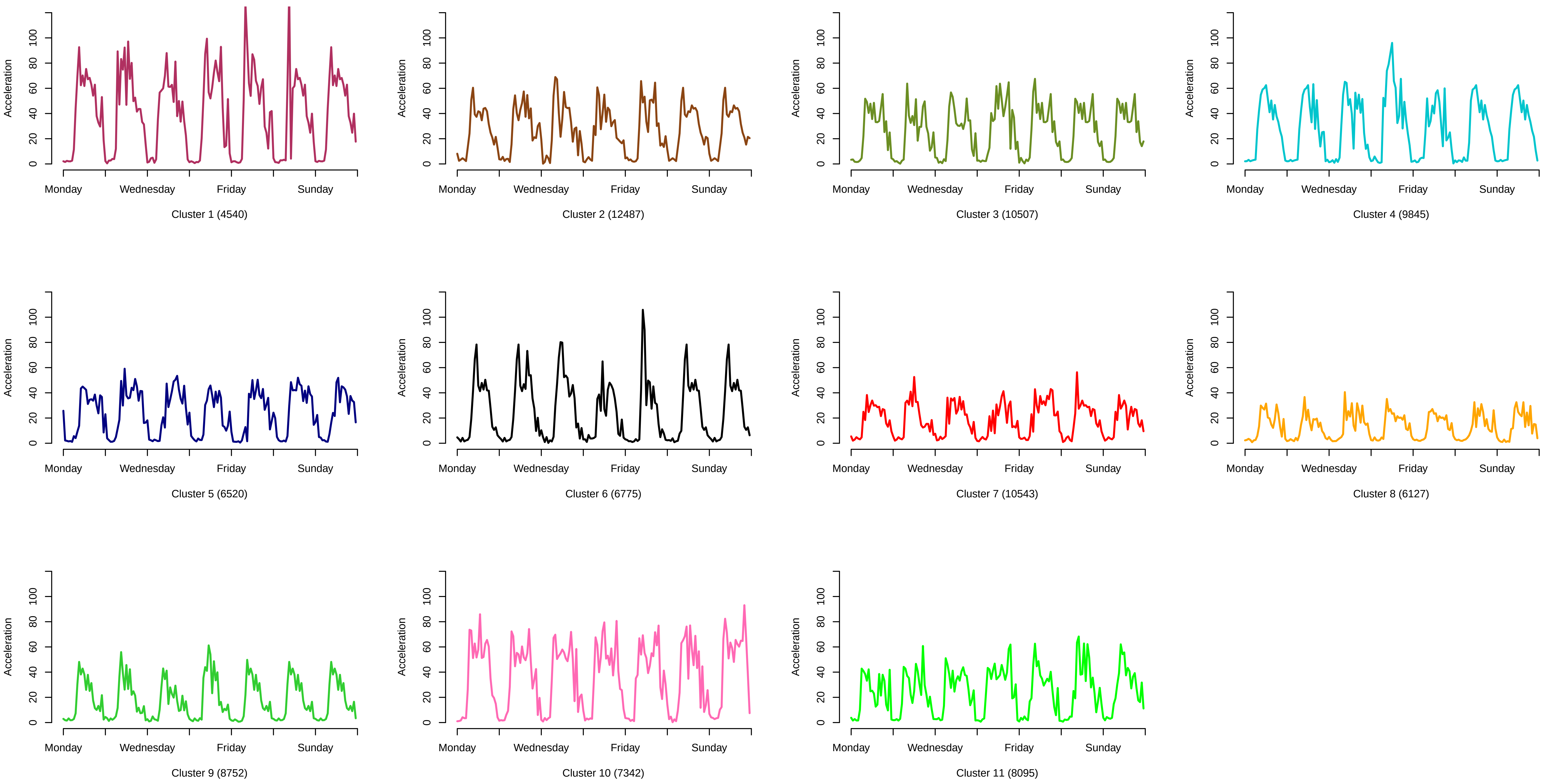
Acceleration
0
20
40
60
80
100
Monday
Wednesday
Friday
Sunday
Cluster 1 (4540)
Cluster 2 (12487)
Cluster 3 (10507)
Cluster 4 (9845)
Cluster 5 (6520)
Cluster 6 (6775)
Cluster 7 (10543)
Cluster 8 (6127)
Cluster 9 (8752)
Cluster 10 (7342)
Cluster 11 (8095)

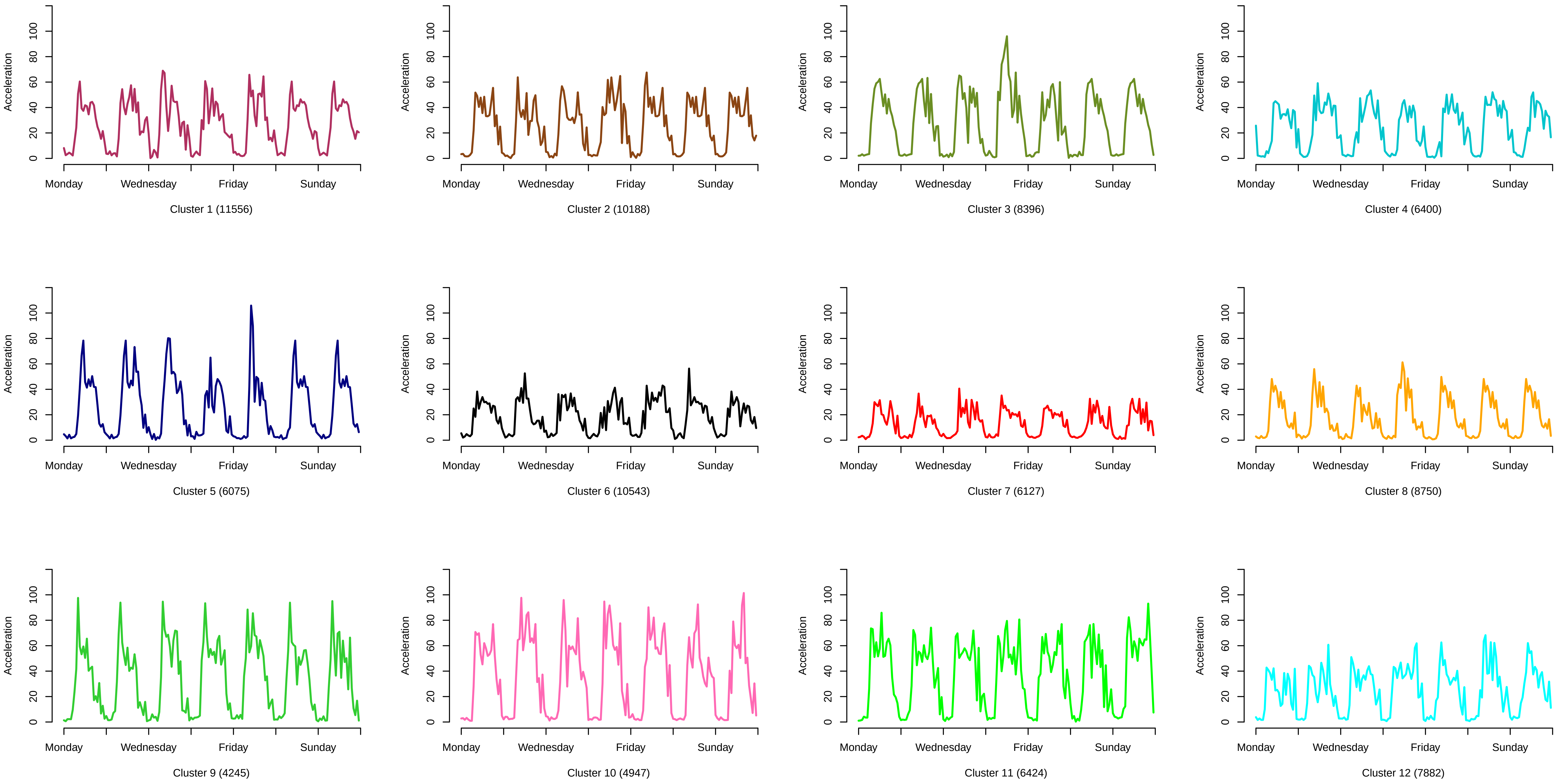
Acceleration
0
20
40
60
80
100
Monday
Wednesday
Friday
Sunday
Cluster 1 (11556)
Cluster 2 (10188)
Cluster 3 (8396)
Cluster 4 (6400)
Cluster 5 (6075)
Cluster 6 (10543)
Cluster 7 (6127)
Cluster 8 (8750)
Cluster 9 (4245)
Cluster 10 (4947)
Cluster 11 (6424)
Cluster 12 (7882)

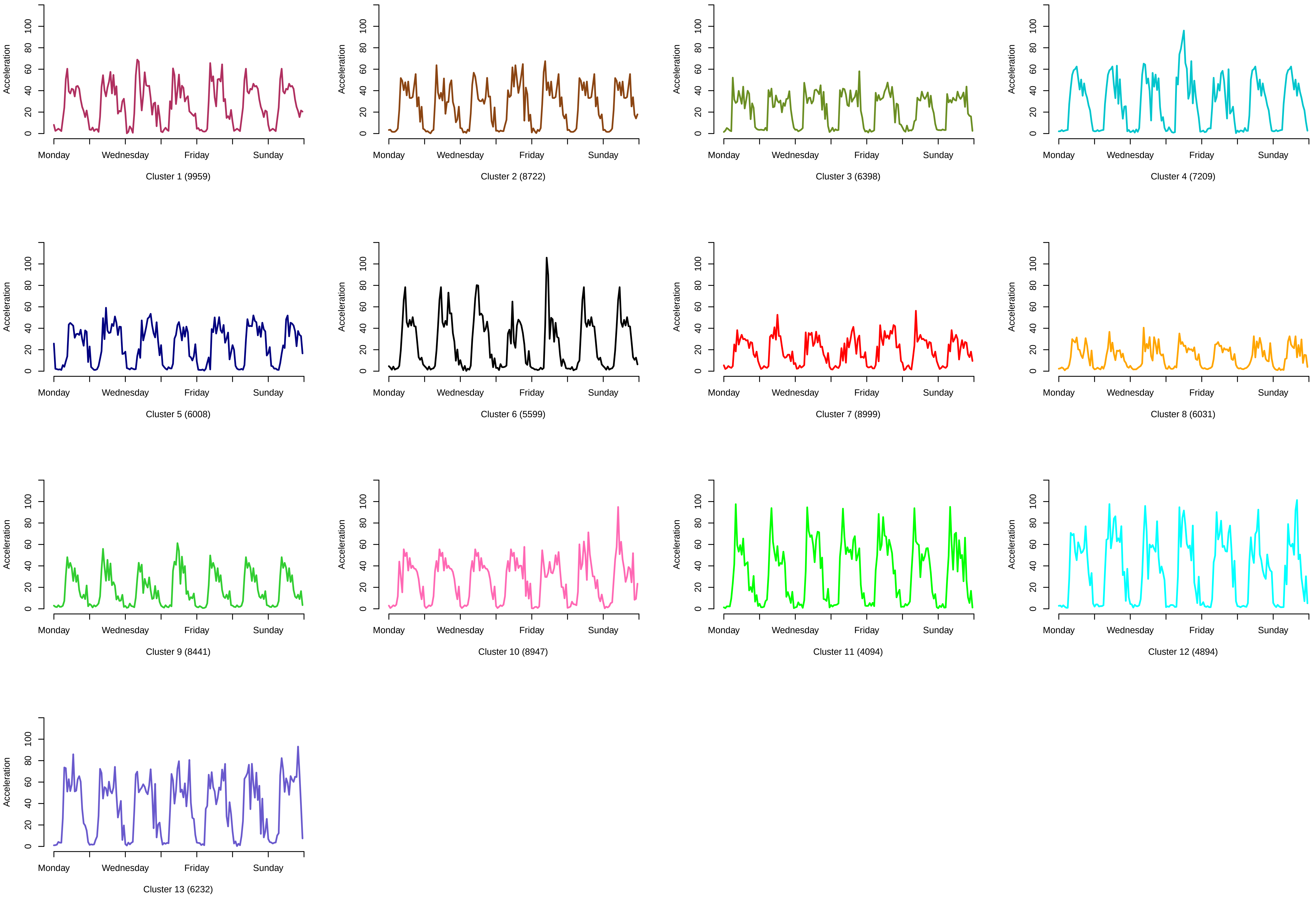
Acceleration
0
20
40
60
80
100
Monday
Wednesday
Friday
Sunday
Cluster 1 (9959)
Cluster 2 (8722)
Cluster 3 (6398)
Cluster 4 (7209)
Cluster 5 (6008)
Cluster 6 (5599)
Cluster 7 (8999)
Cluster 8 (6031)
Cluster 9 (8441)
Cluster 10 (8947)
Cluster 11 (4094)
Cluster 12 (4894)
Cluster 13 (6232)

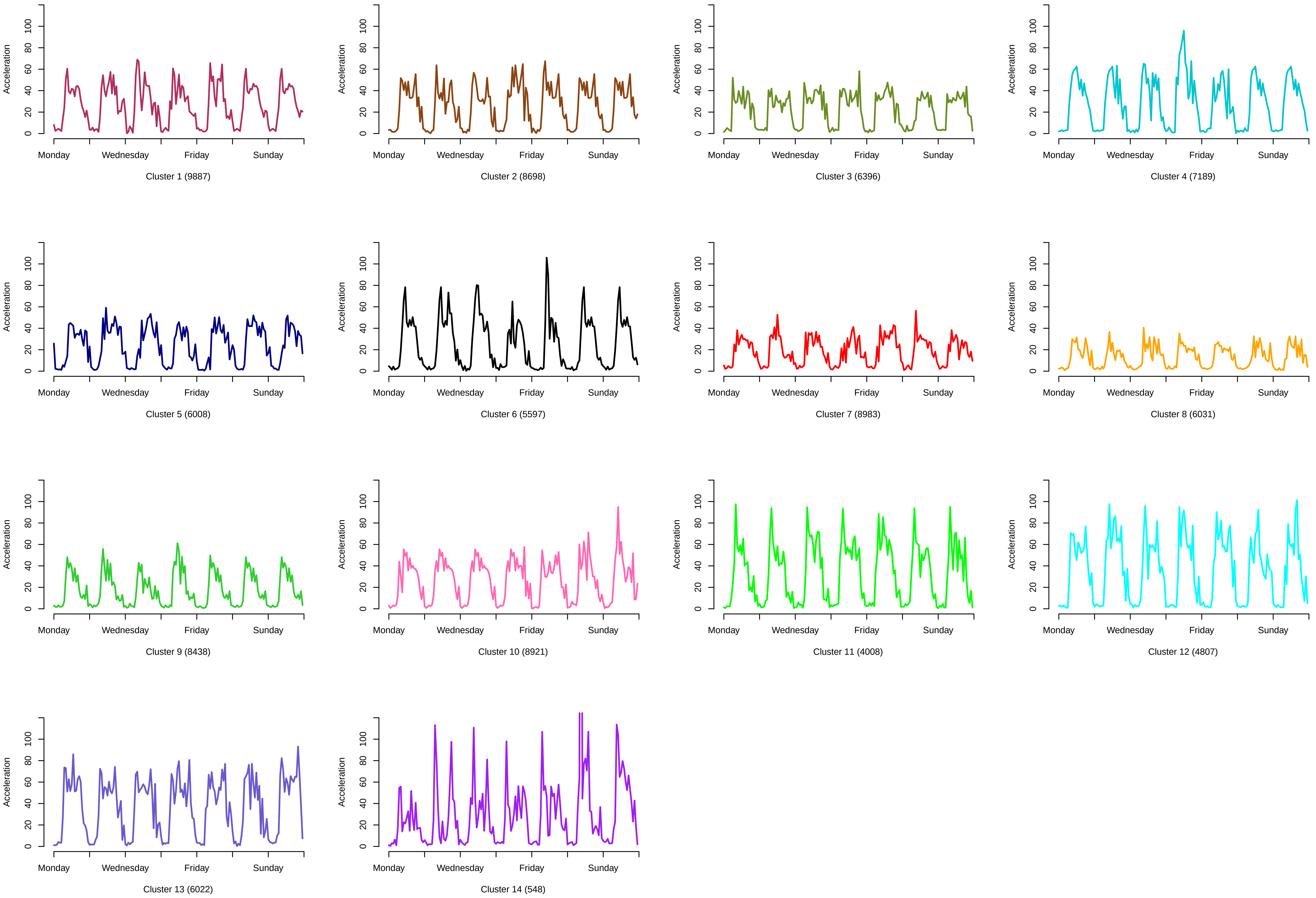
Acceleration
0
20
40
60
80
100
Monday
Wednesday
Friday
Sunday
Cluster 1 (9887)
Cluster 2 (8698)
Cluster 3 (6396)
Cluster 4 (7189)
Cluster 5 (6008)
Cluster 6 (5597)
Cluster 7 (8983)
Cluster 8 (6031)
Cluster 9 (8438)
Cluster 10 (8921)
Cluster 11 (4008)
Cluster 12 (4807)
Cluster 13 (6022)
Cluster 14 (548)

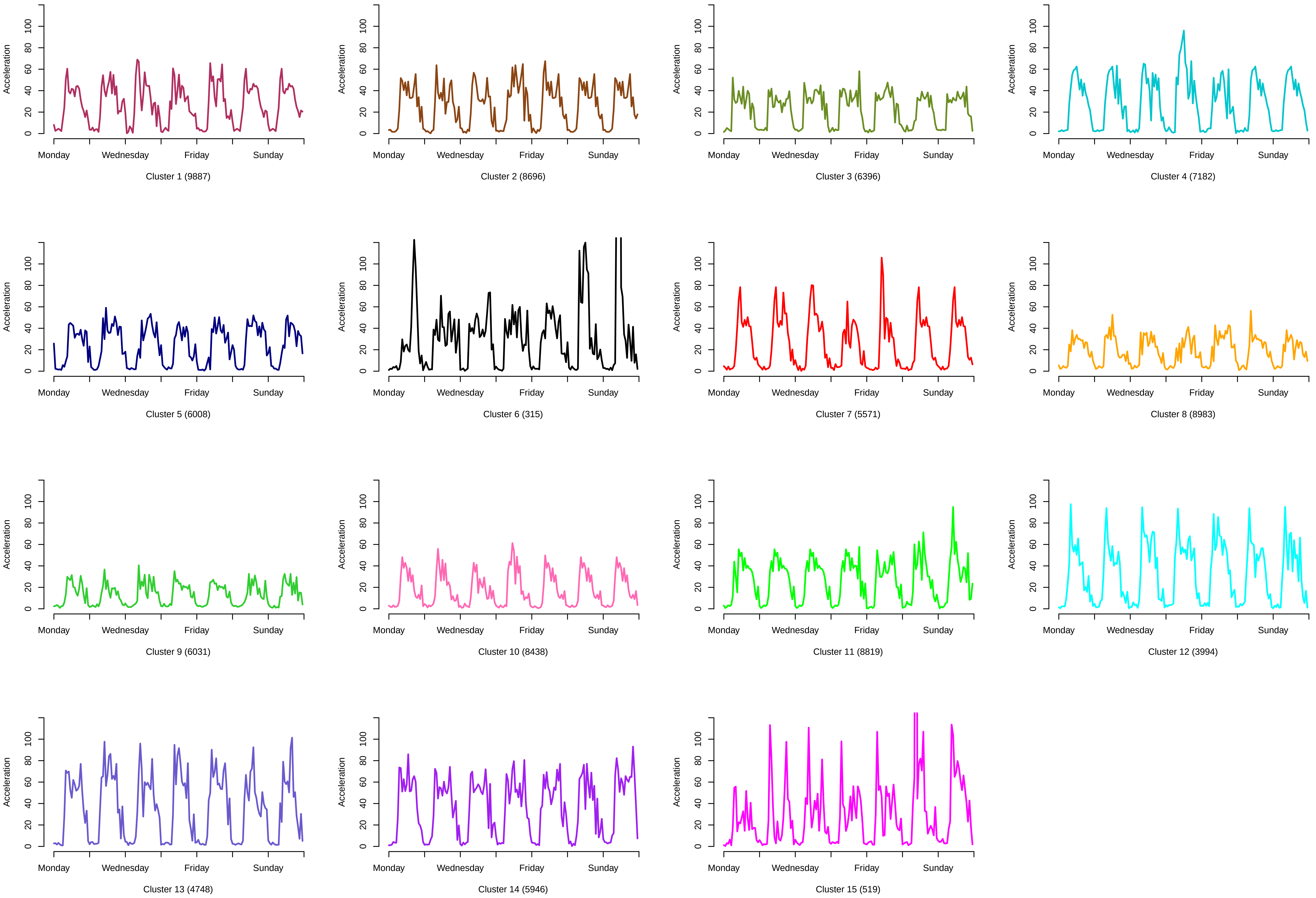
Acceleration
0
20
40
60
80
100
Monday
Wednesday
Friday
Sunday
Cluster 1 (9887)
Cluster 2 (8696)
Cluster 3 (6396)
Cluster 4 (7182)
Cluster 5 (6008)
Cluster 6 (315)
Cluster 7 (5571)
Cluster 8 (8983)
Cluster 9 (6031)
Cluster 10 (8438)
Cluster 11 (8819)
Cluster 12 (3994)
Cluster 13 (4748)
Cluster 14 (5946)
Cluster 15 (519)

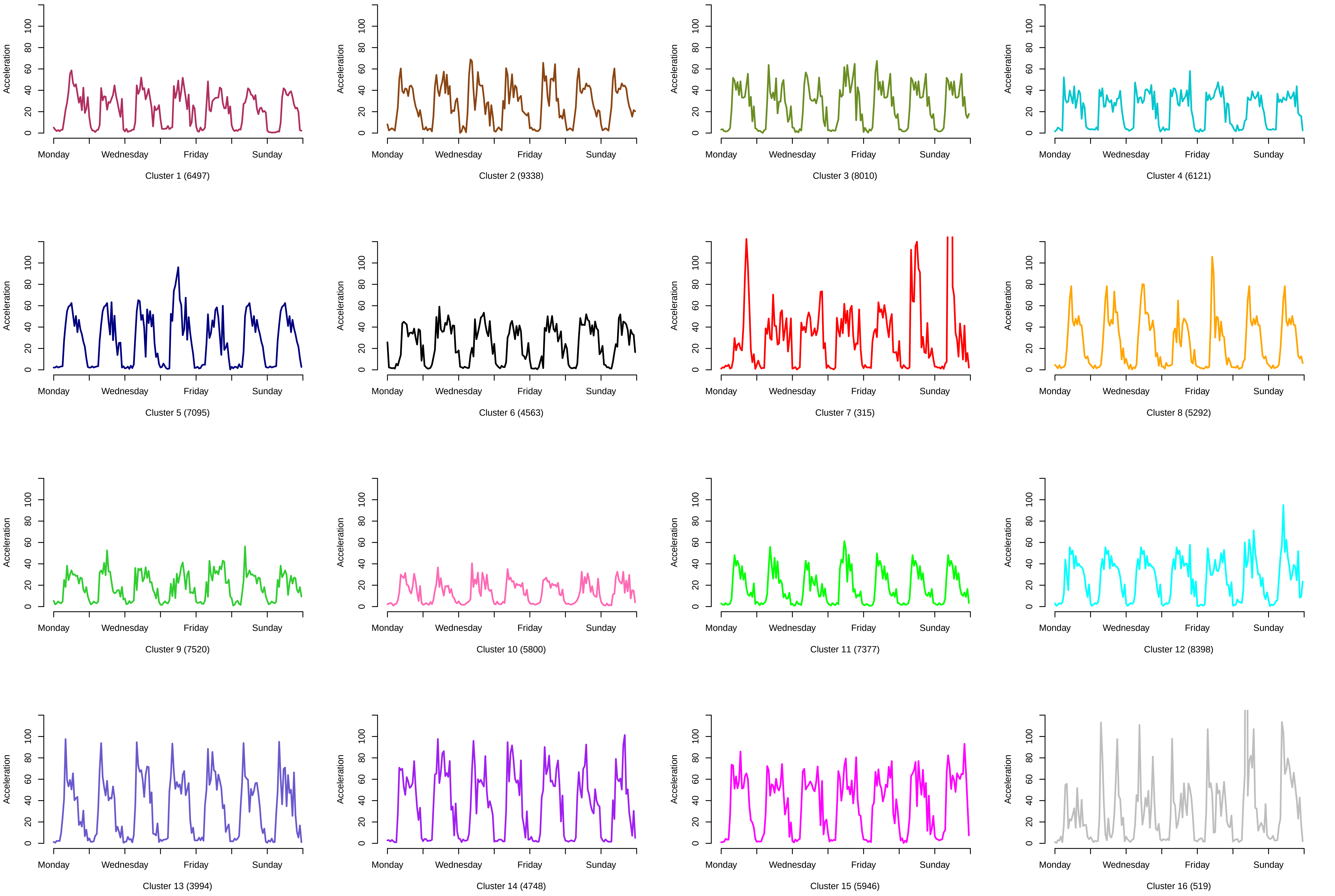
Acceleration
0
20
40
60
80
100
Monday
Wednesday
Friday
Sunday
Cluster 1 (6497)
Cluster 2 (9338)
Cluster 3 (8010)
Cluster 4 (6121)
Cluster 5 (7095)
Cluster 6 (4563)
Cluster 7 (315)
Cluster 8 (5292)
Cluster 9 (7520)
Cluster 10 (5800)
Cluster 11 (7377)
Cluster 12 (8398)
Cluster 13 (3994)
Cluster 14 (4748)
Cluster 15 (5946)
Cluster 16 (519)
