# Supplementary material for: Clustering Accelerometer Activity Patterns from the UK Biobank Cohort
Source: Sensors (Basel). 2021 Dec 9;21(24):8220. doi: 10.3390/s21248220 (PMC8709415; doi:10.3390/s21248220)
Supplement: Supplementary file 1 [file sensors-21-08220-s001.zip › SupplementaryTables S1toS15.pdf]

Table S1: Gender (n=91533)	Active 9 to 5	Active	Morning Movers	Get up and Active	Live for the Weekend	Moderates	Leisurely 9 to 5	Sedate	Inactive	Classification sample	Wearable sample	UK Biobank
Female	61.1%	60.2%	59.5%	61.2%	56.0%	58.2%	49.5%	52.8%	45.3%	56.5%	56.2%	54.4%
Male	38.9%	39.8%	40.5%	38.8%	44.0%	41.8%	50.5%	47.2%	54.7%	43.5%	43.8%	45.6%

Table S2: Age (n=91533)	Active 9 to 5	Active	Morning Movers	Get up and Active	Live for the Weekend	Moderates	Leisurely 9 to 5	Sedate	Inactive			
40 to 44	22.4%	11.8%	6.9%	7.3%	13.5%	8.2%	18.6%	2.9%	5.4%	10.1%	10.4%	10.4%
45 to 49	24.1%	15.9%	10.5%	10.0%	17.6%	11.8%	23.2%	5.2%	8.6%	13.3%	13.3%	13.2%
50 to 54	21.3%	18.7%	15.2%	14.3%	18.5%	15.8%	24.3%	9.9%	12.2%	16.2%	15.4%	15.3%
55 to 59	16.3%	23.6%	24.1%	21.1%	18.9%	21.8%	16.6%	19.6%	18.3%	20.1%	18.5%	18.2%
60 to 64	11.5%	22.4%	29.2%	29.1%	20.3%	26.7%	10.8%	34.8%	28.7%	24.8%	24.3%	24.3%
65 to 69	4.5%	7.6%	14.1%	18.2%	11.2%	15.7%	6.6%	27.6%	26.8%	15.5%	18.1%	18.7%

Table S3: Ethnicity (n=91533)	Active 9 to 5	Active	Morning Movers	Get up and Active	Live for the Weekend	Moderates	Leisurely 9 to 5	Sedate	Inactive			
White/British	95.4%	97.2%	97.5%	97.2%	96.3%	95.9%	94.1%	98.0%	96.5%	96.6%	96.4%	94.1%
Mixed	0.8%	0.5%	0.5%	0.5%	0.6%	0.6%	0.8%	0.3%	0.5%	0.5%	0.6%	0.6%
South Asian	0.9%	0.3%	0.6%	0.5%	0.8%	0.8%	1.7%	0.5%	1.0%	0.8%	0.8%	1.6%
Black	1.2%	0.8%	0.4%	0.8%	0.9%	1.0%	1.8%	0.4%	0.7%	0.8%	0.9%	1.6%
Other	1.4%	1.0%	0.7%	0.8%	1.0%	1.3%	1.2%	0.5%	0.8%	0.9%	1.0%	1.6%
PNA/DK	0.3%	0.3%	0.3%	0.3%	0.4%	0.3%	0.3%	0.4%	0.5%	0.3%	0.4%	0.6%

Table S4: Employment (n=91533)	Active 9 to 5	Active	Morning Movers	Get up and Active	Live for the Weekend	Moderates	Leisurely 9 to 5	Sedate	Inactive			
None/PNA	0.5%	0.9%	0.8%	0.8%	0.6%	0.6%	0.5%	0.6%	0.8%	0.7%	0.7%	1.1%
In paid employment or self-employed	81.6%	65.3%	55.8%	56.3%	71.1%	59.2%	85.8%	44.7%	47.8%	61.8%	62.3%	57.4%
Retired	11.6%	25.1%	36.4%	37.0%	23.4%	32.4%	10.0%	49.1%	40.9%	31.1%	30.6%	33.0%
Looking after home and/or family	3.9%	5.2%	4.0%	3.1%	2.4%	3.0%	1.2%	1.9%	1.7%	2.8%	2.9%	2.8%
Unable to work because of sickness or disability	0.5%	1.1%	1.2%	1.0%	0.8%	2.4%	0.7%	2.0%	6.1%	1.6%	1.7%	3.4%
Unemployed	1.1%	1.4%	1.0%	0.8%	1.0%	1.5%	1.3%	0.9%	1.9%	1.2%	1.2%	1.7%
Doing unpaid or voluntary work	0.3%	0.8%	0.5%	0.7%	0.4%	0.6%	0.3%	0.6%	0.5%	0.5%	0.5%	0.5%
Full or part-time student	0.5%	0.3%	0.2%	0.3%	0.3%	0.2%	0.3%	0.2%	0.3%	0.3%	0.3%	0.3%

**Table S5: Mode (Excl None) (n=51667)**	Active 9 to 5	Active	Morning Movers	Get up and Active	Live for the Weekend	Moderates	Leisurely 9 to 5	Sedate	Inactive			
Car/motor vehicle	53.4%	59.4%	63.5%	58.7%	60.8%	60.7%	58.1%	65.6%	63.7%	60.1%	60.0%	63.0%
Walk/cycle	11.8%	11.0%	8.0%	8.7%	7.2%	6.6%	6.4%	6.3%	5.3%	8.0%	7.9%	7.6%
Public transport	7.4%	6.1%	6.3%	8.1%	7.6%	9.0%	10.5%	8.3%	10.0%	8.2%	8.3%	9.0%
Mixed	27.4%	23.5%	22.1%	24.5%	24.3%	23.7%	24.9%	19.8%	21.0%	23.8%	23.8%	20.4%
PNA/DK	23.8%	40.9%	49.4%	49.1%	33.9%	47.1%	18.2%	60.3%	58.2%	43.6%	43.1%	47.2%

**Table S6: Education (n=91533)**	Active 9 to 5	Active	Morning Movers	Get up and Active	Live for the Weekend	Moderates	Leisurely 9 to 5	Sedate	Inactive			
College/University	46.9%	41.9%	39.2%	44.3%	46.1%	44.8%	46.5%	37.4%	39.6%	42.9%	43.1%	32.1%
A levels	13.6%	13.8%	12.4%	13.3%	13.4%	13.5%	13.7%	12.3%	12.7%	13.1%	13.1%	11.0%
GCSE	19.8%	21.9%	21.6%	19.9%	20.2%	19.5%	19.4%	21.4%	19.5%	20.4%	20.3%	20.9%
CSE	5.9%	4.9%	4.7%	3.0%	3.9%	3.8%	5.0%	3.1%	3.0%	4.0%	4.0%	5.4%
NVQ	4.6%	4.8%	5.3%	4.7%	5.0%	5.1%	5.5%	6.6%	6.4%	5.3%	5.3%	6.5%
Other	3.6%	4.7%	5.3%	5.6%	4.5%	4.6%	4.1%	6.3%	5.3%	5.0%	5.0%	5.1%
None	4.9%	7.2%	10.4%	8.2%	6.0%	7.7%	4.8%	11.8%	12.1%	8.2%	8.1%	16.9%
PNA	0.7%	0.7%	1.0%	1.0%	0.9%	1.0%	1.1%	1.2%	1.3%	1.0%	1.0%	2.0%

**Table S7: Income (n=91533)**	Active 9 to 5	Active	Morning Movers	Get up and Active	Live for the Weekend	Moderates	Leisurely 9 to 5	Sedate	Inactive			
PNA/DK	8.2%	10.9%	11.6%	11.3%	8.7%	10.4%	7.5%	11.9%	10.8%	10.3%	10.3%	15.3%
Less than 18,000	8.5%	12.2%	13.5%	13.0%	9.8%	15.2%	7.7%	17.3%	21.6%	13.2%	13.1%	19.3%
18,000 to 30,999	17.3%	20.6%	22.6%	22.8%	19.1%	22.7%	17.5%	26.1%	24.0%	21.7%	21.5%	21.5%
31,000 to 51,999	27.5%	26.2%	26.4%	25.6%	26.3%	25.3%	27.9%	24.1%	22.9%	25.8%	25.7%	22.1%
52,000 to 100,000	29.5%	23.3%	20.4%	21.0%	27.3%	20.8%	29.6%	16.9%	16.2%	22.5%	22.7%	17.2%
Greater than 100,000	9.1%	6.8%	5.5%	6.3%	8.8%	5.6%	9.8%	3.7%	4.4%	6.5%	6.7%	4.6%

Table S8: Obesity (n=91533)	Active 9 to 5	Active	Morning Movers	Get up and Active	Live for the Weekend	Moderates	Leisurely 9 to 5	Sedate	Inactive			
Underweight	1.2%	1.0%	0.6%	0.6%	0.6%	0.4%	0.4%	0.3%	0.4%	0.6%	0.6%	0.5%
Healthy	55.8%	54.1%	43.5%	42.5%	41.1%	33.6%	33.7%	27.2%	20.1%	38.7%	38.6%	32.3%
Overweight	34.2%	36.4%	42.0%	41.7%	41.8%	43.0%	41.2%	45.0%	38.7%	41.1%	41.0%	42.2%
Obese	8.6%	8.4%	13.9%	15.1%	16.4%	22.8%	24.6%	27.2%	40.2%	19.4%	19.6%	24.3%
NA	0.2%	0.1%	0.1%	0.1%	0.2%	0.3%	0.2%	0.3%	0.7%	0.2%	0.2%	0.6%

Table S9: Overall health (n=91533)	Active 9 to 5	Active	Morning Movers	Get up and Active	Live for the Weekend	Moderates	Leisurely 9 to 5	Sedate	Inactive			
PNA/DNK	0.2%	0.2%	0.2%	0.2%	0.2%	0.2%	0.3%	0.3%	0.5%	0.2%	0.2%	0.7%
Excellent	31.3%	28.9%	24.9%	22.9%	24.2%	18.1%	20.1%	15.4%	11.1%	21.7%	21.7%	16.3%
Good	58.5%	59.7%	61.6%	62.0%	60.8%	59.3%	60.3%	61.1%	49.8%	59.8%	59.7%	57.5%
Fair	9.2%	10.2%	11.8%	13.4%	13.4%	18.8%	17.0%	19.6%	29.4%	15.7%	15.8%	21.0%
Poor	0.7%	0.9%	1.6%	1.5%	1.4%	3.6%	2.3%	3.5%	9.3%	2.6%	2.6%	4.5%

Table S10: Long standing illness (n=91533)	Active 9 to 5	Active	Morning Movers	Get up and Active	Live for the Weekend	Moderates	Leisurely 9 to 5	Sedate	Inactive			
PNA/DNK	1.5%	1.7%	1.9%	1.7%	1.9%	2.1%	1.9%	2.4%	2.1%	1.9%	1.9%	2.7%
No	81.5%	77.7%	74.6%	71.4%	74.9%	66.3%	72.7%	62.5%	48.1%	70.1%	70.1%	65.6%
Yes	17.1%	20.6%	23.4%	26.9%	23.1%	31.6%	25.4%	35.1%	49.8%	28.0%	28.0%	31.8%

Table S11: Getting up (n=91533)	Active 9 to 5	Active	Morning Movers	Get up and Active	Live for the Weekend	Moderates	Leisurely 9 to 5	Sedate	Inactive			
Not at all easy	2.2%	3.3%	2.5%	2.3%	2.4%	6.5%	2.8%	2.8%	6.3%	3.3%	3.4%	3.9%
Not very easy	12.4%	15.1%	11.5%	12.0%	12.4%	20.4%	13.8%	12.1%	17.8%	13.8%	13.9%	13.9%
Fairly easy	52.8%	52.8%	51.9%	49.9%	51.8%	50.3%	50.1%	50.0%	46.0%	50.6%	50.7%	49.1%
Very easy	32.2%	28.4%	33.5%	35.2%	32.8%	22.1%	32.6%	34.4%	29.1%	31.6%	31.4%	31.9%
PNA/DK	0.5%	0.5%	0.6%	0.6%	0.6%	0.7%	0.7%	0.7%	0.8%	0.6%	0.6%	1.1%

**Table S12: Chronotype (n=91533)**	Active 9 to 5	Active	Morning Movers	Get up and Active	Live for the Weekend	Moderates	Leisurely 9 to 5	Sedate	Inactive			
Definitely a 'morning' person	30.7%	19.0%	24.4%	25.0%	26.4%	12.1%	28.7%	21.6%	17.0%	22.9%	22.9%	23.9%
More a 'morning' than 'evening' person	35.1%	33.5%	35.7%	35.5%	35.3%	26.7%	34.4%	35.0%	27.6%	33.5%	33.4%	31.3%
More an 'evening' than a 'morning' person	20.3%	26.5%	23.8%	22.3%	22.1%	33.5%	20.5%	24.7%	29.0%	24.6%	24.7%	25.1%
Definitely an 'evening' person	4.5%	9.1%	5.5%	6.1%	5.6%	16.7%	6.5%	7.4%	15.7%	8.2%	8.3%	8.0%
PNA/DK	9.5%	11.8%	10.6%	11.1%	10.6%	11.0%	9.8%	11.3%	10.8%	10.8%	10.8%	11.6%

**Table S13: Deprivation (n=91427)**	Active 9 to 5	Active	Morning Movers	Get up and Active	Live for the Weekend	Moderates	Leisurely 9 to 5	Sedate	Inactive			
Least deprived	21.7%	24.5%	24.5%	24.3%	23.5%	20.9%	19.7%	24.1%	19.2%	22.8%	22.6%	20.0%
Second least deprived	21.7%	22.0%	23.2%	23.0%	21.7%	20.7%	19.4%	22.8%	18.9%	21.7%	21.6%	20.0%
Middle deprived	20.0%	21.6%	21.2%	20.7%	21.0%	20.0%	20.4%	21.0%	18.6%	20.6%	20.6%	20.0%
Second most deprived	20.5%	18.4%	18.6%	18.6%	19.7%	20.3%	21.9%	18.0%	21.8%	19.5%	19.6%	20.0%
Most deprived	16.0%	13.4%	12.4%	13.3%	14.0%	18.0%	18.4%	14.0%	21.3%	15.2%	15.4%	20.0%
NA	0.1%	0.1%	0.1%	0.1%	0.1%	0.1%	0.2%	0.2%	0.2%	0.1%	0.1%	0.1%

**Table S14: Season (n=91533)**	Active 9 to 5	Active	Morning Movers	Get up and Active	Live for the Weekend	Moderates	Leisurely 9 to 5	Sedate	Inactive		
Spring	25.1%	24.7%	21.6%	23.3%	23.5%	21.0%	23.1%	20.3%	20.5%	22.5%	22.8%
Summer	29.3%	27.7%	27.9%	25.1%	27.5%	26.0%	26.6%	24.1%	24.4%	26.4%	26.5%
Autumn	27.8%	30.1%	29.9%	30.1%	28.6%	29.7%	29.1%	30.4%	30.1%	29.6%	29.7%
Winter	17.8%	17.5%	20.6%	21.5%	20.4%	23.3%	21.2%	25.2%	24.9%	21.6%	21.1%

**Table S15: Assessment centre (n=91533)**	Active 9 to 5	Active	Morning Movers	Get up and Active	Live for the Weekend	Moderates	Leisurely 9 to 5	Sedate	Inactive			
North	34.7%	41.5%	40.4%	37.1%	35.8%	39.1%	35.9%	41.3%	38.9%	38.2%	38.2%	42.1%
Midlands	15.0%	14.9%	16.2%	14.9%	15.4%	13.8%	14.6%	15.1%	14.4%	15.0%	14.9%	15.7%
Southern England	22.3%	18.7%	19.3%	21.5%	21.9%	17.8%	21.8%	19.9%	19.1%	20.4%	20.3%	17.2%
London	17.8%	13.2%	13.8%	16.4%	16.8%	18.4%	18.9%	13.9%	16.8%	16.2%	16.4%	13.7%
Wales	3.4%	3.9%	3.9%	3.4%	3.9%	3.9%	3.2%	3.9%	4.2%	3.7%	3.7%	4.2%
Scotland	6.9%	7.6%	6.4%	6.7%	6.1%	7.1%	5.6%	6.0%	6.6%	6.5%	6.5%	7.1%
