# Supplementary figures and images for: Clustering Accelerometer Activity Patterns from the UK Biobank Cohort
Source: Sensors (Basel). 2021 Dec 9;21(24):8220. doi: 10.3390/s21248220 (PMC8709415; doi:10.3390/s21248220)

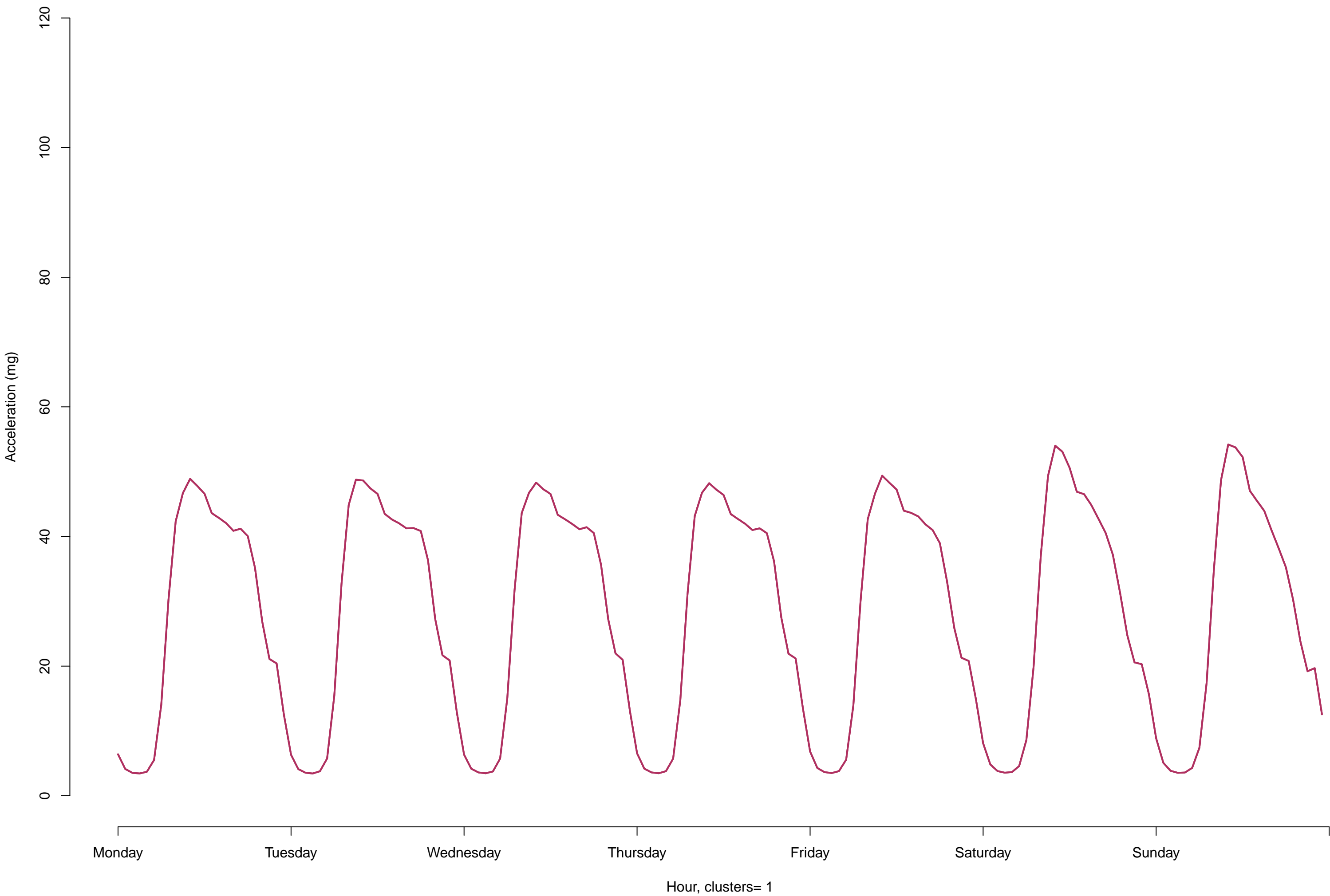

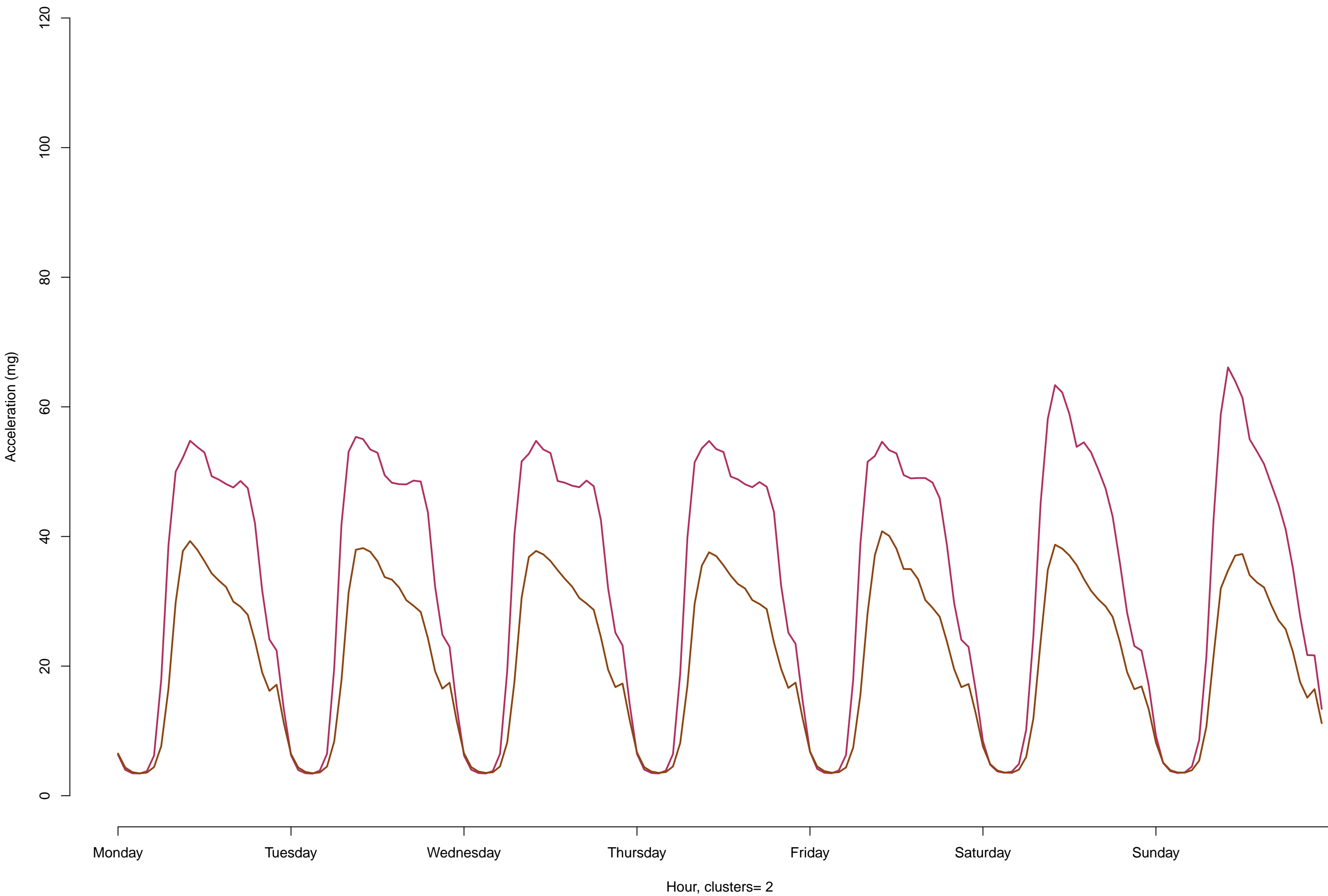

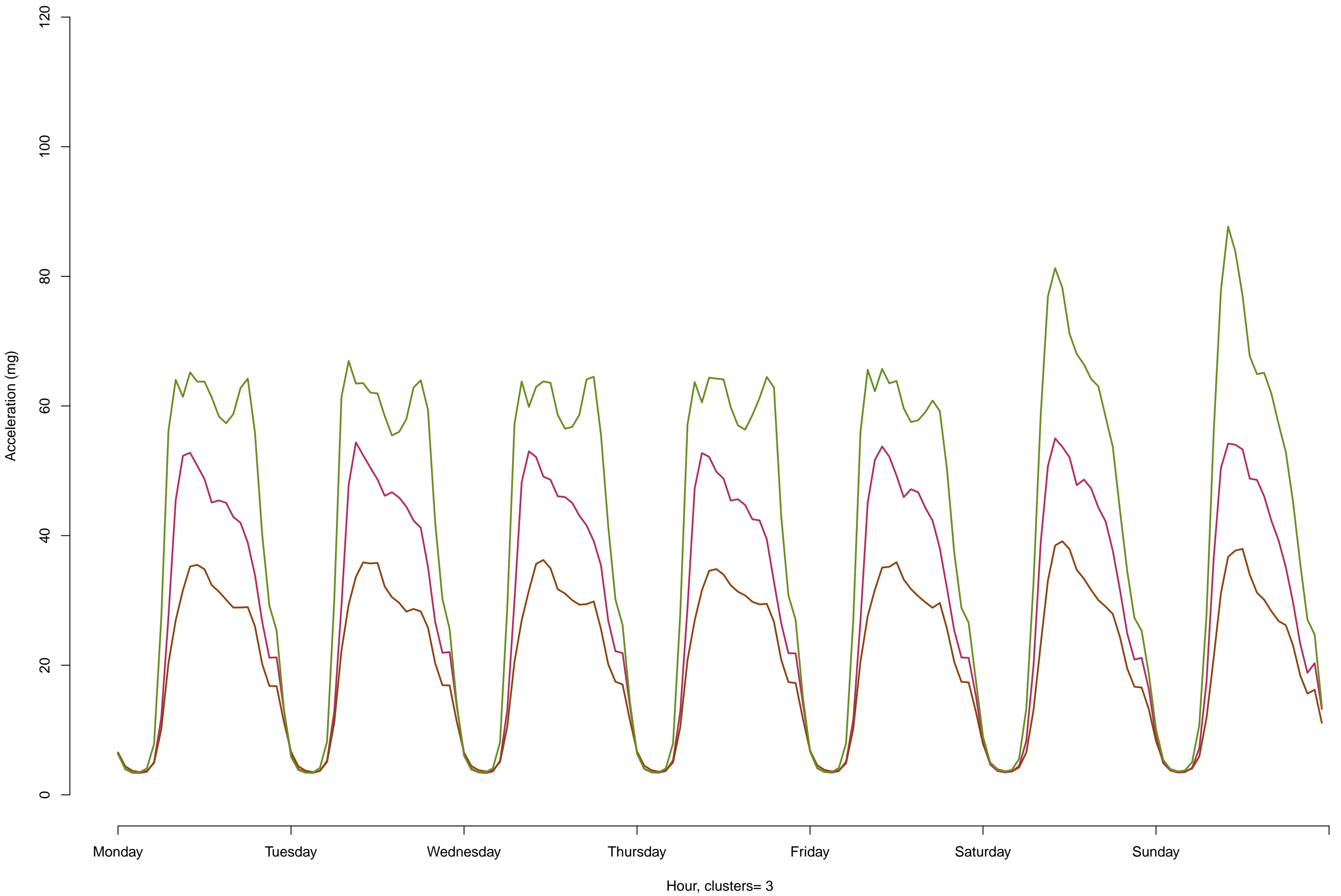

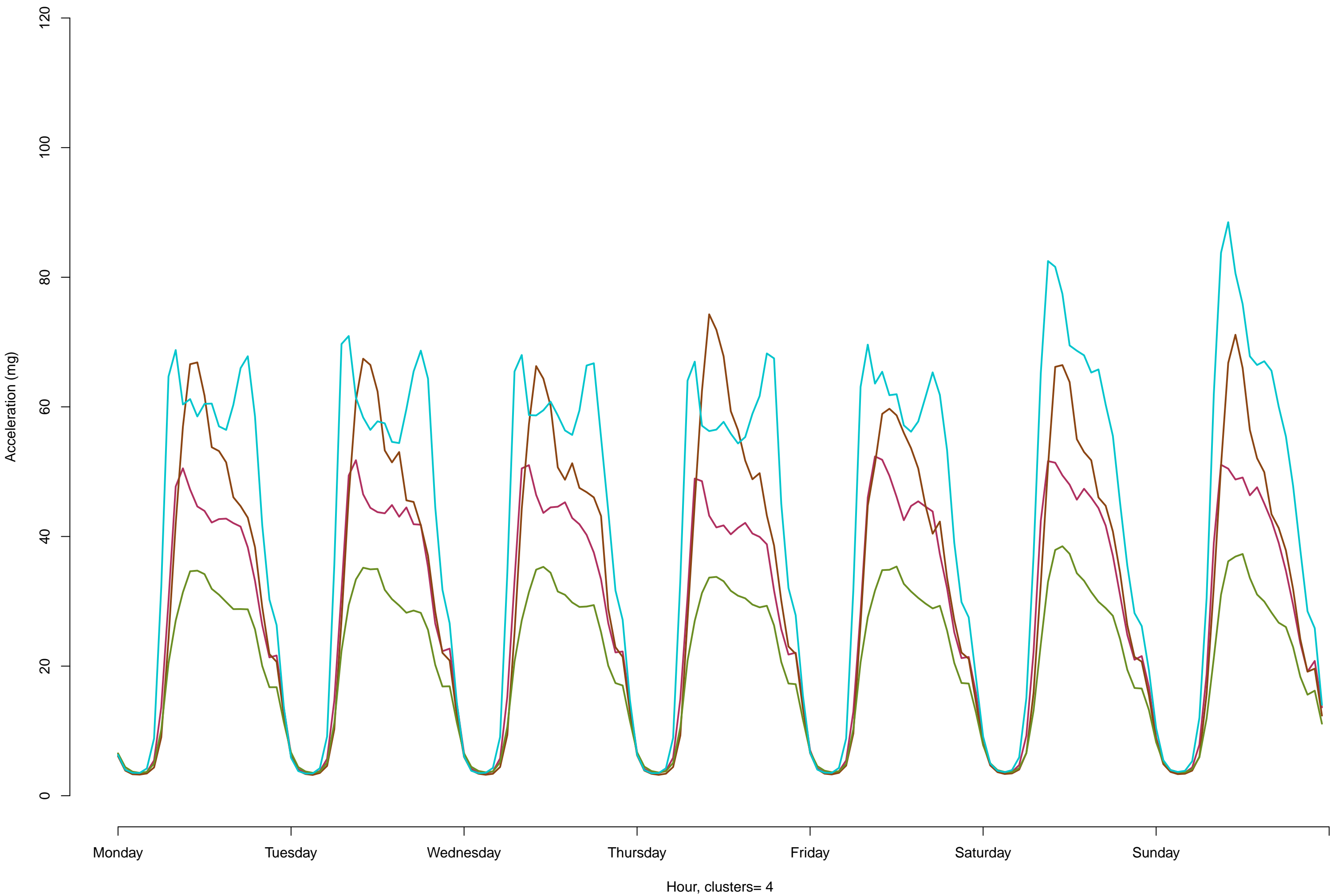

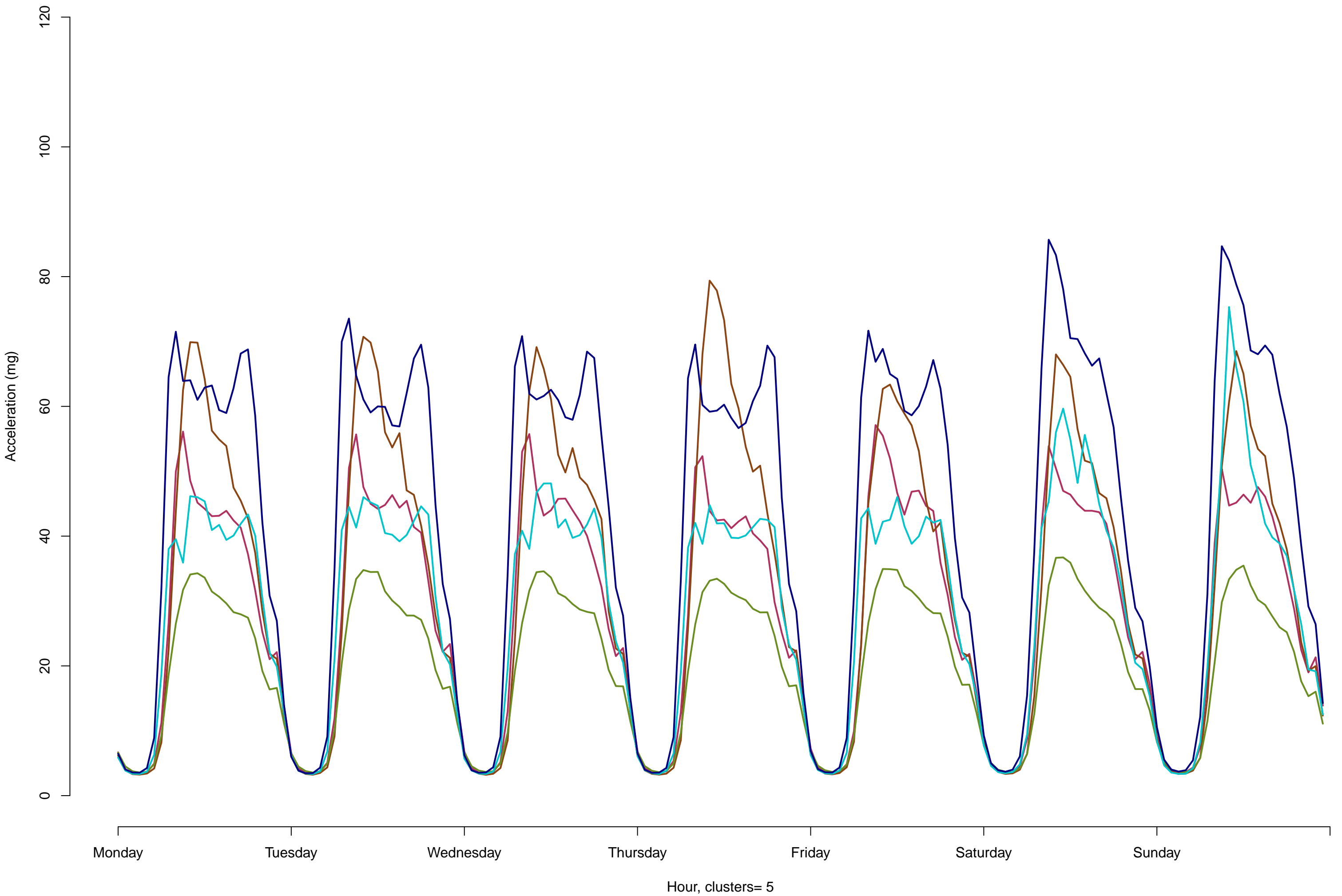

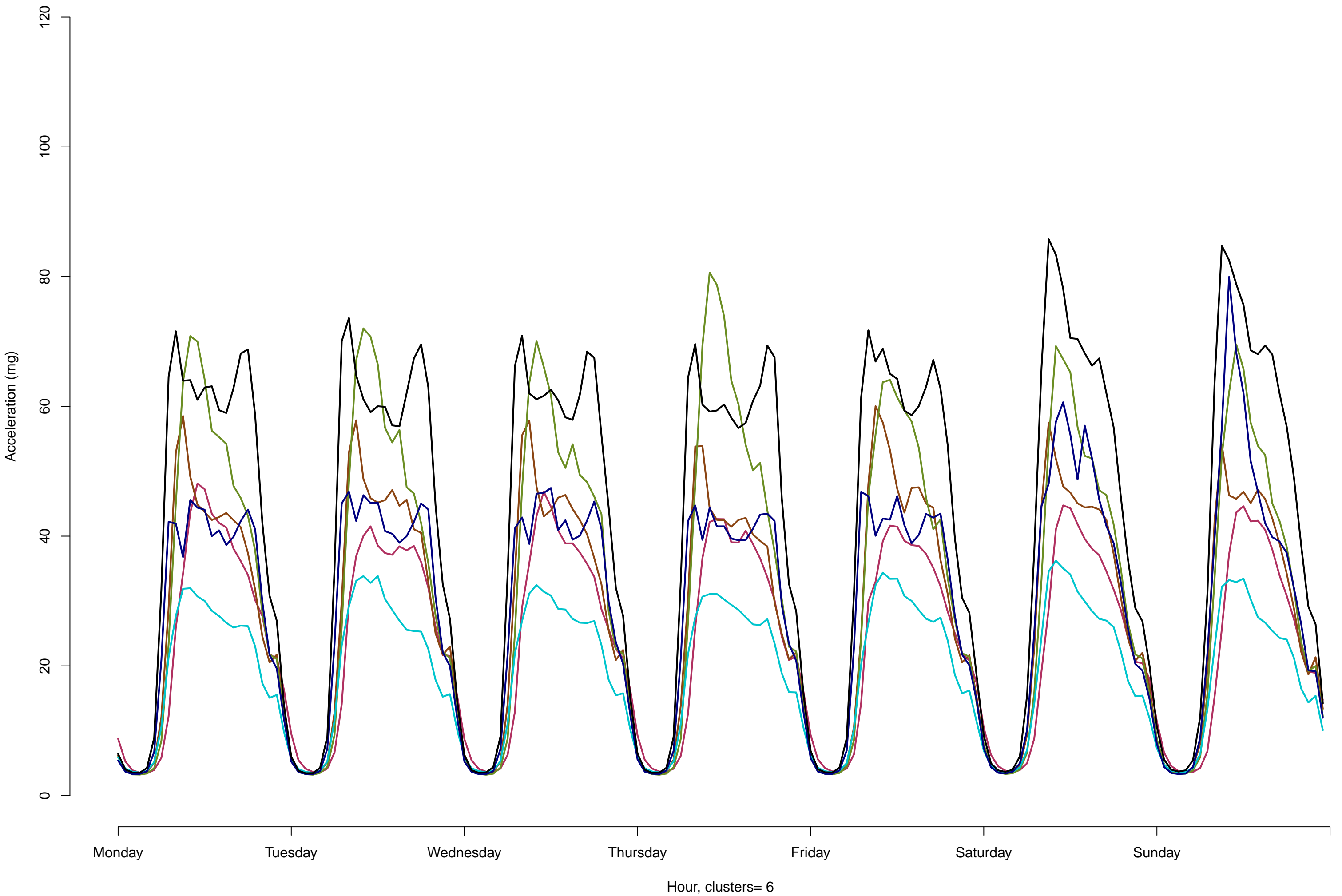

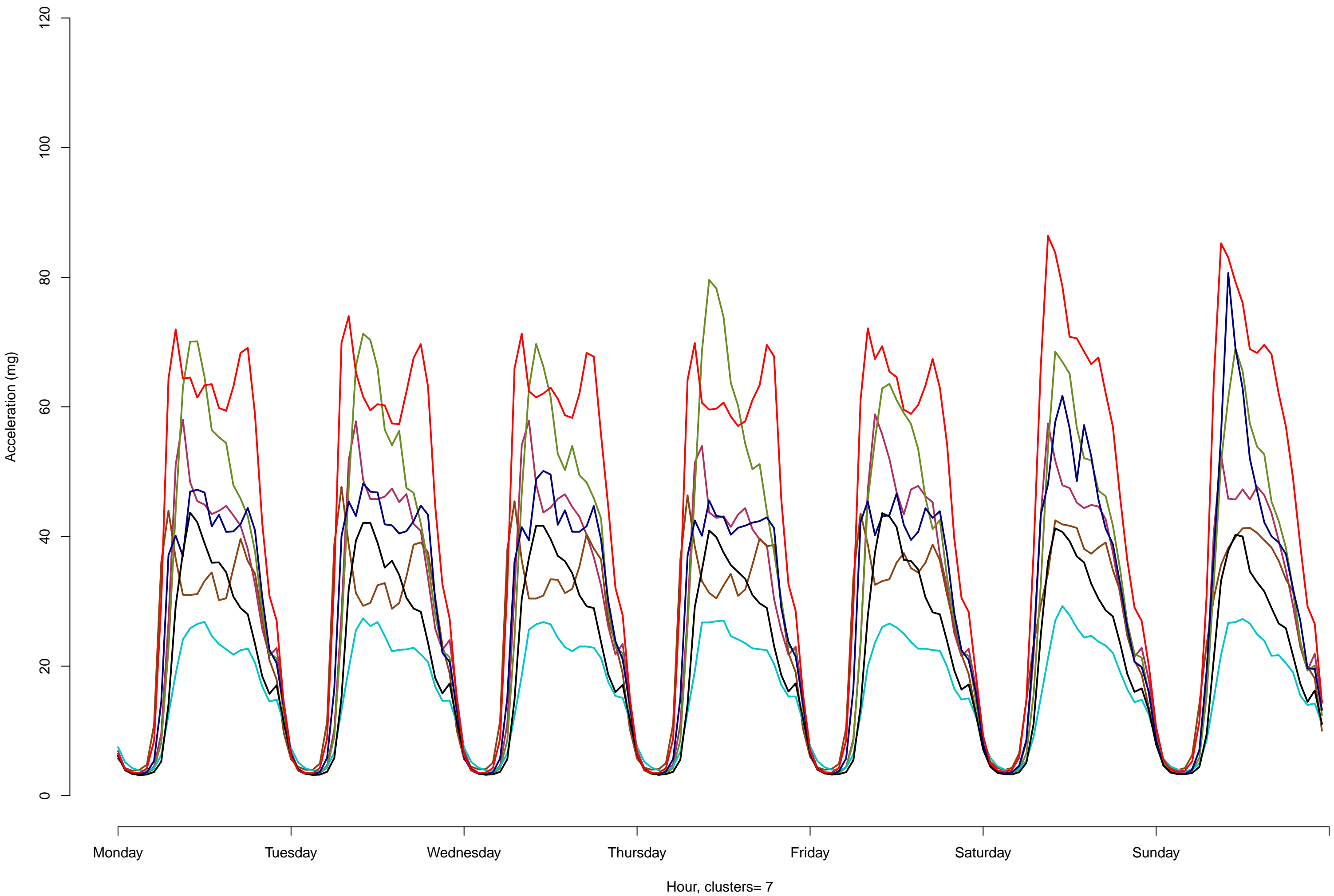

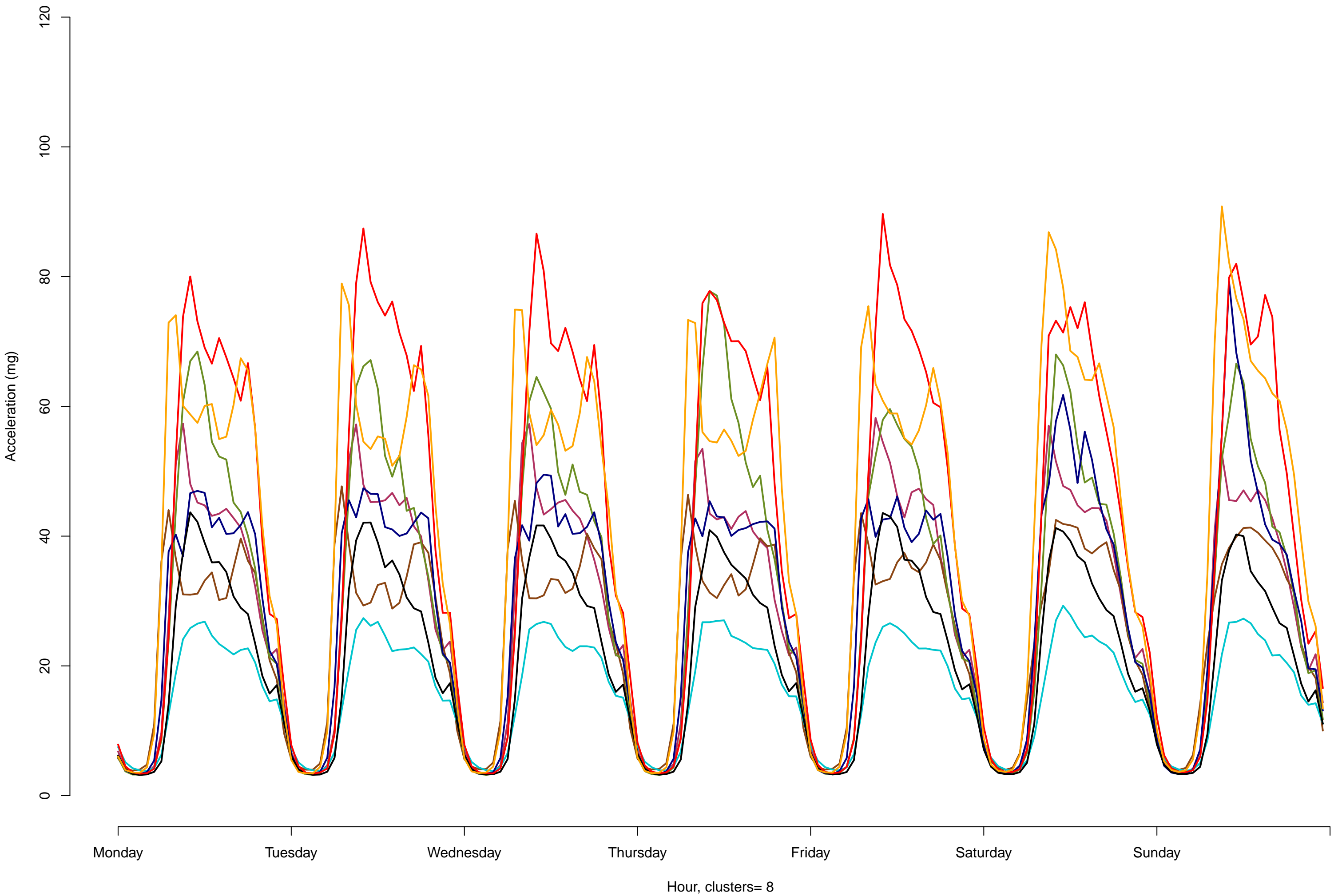

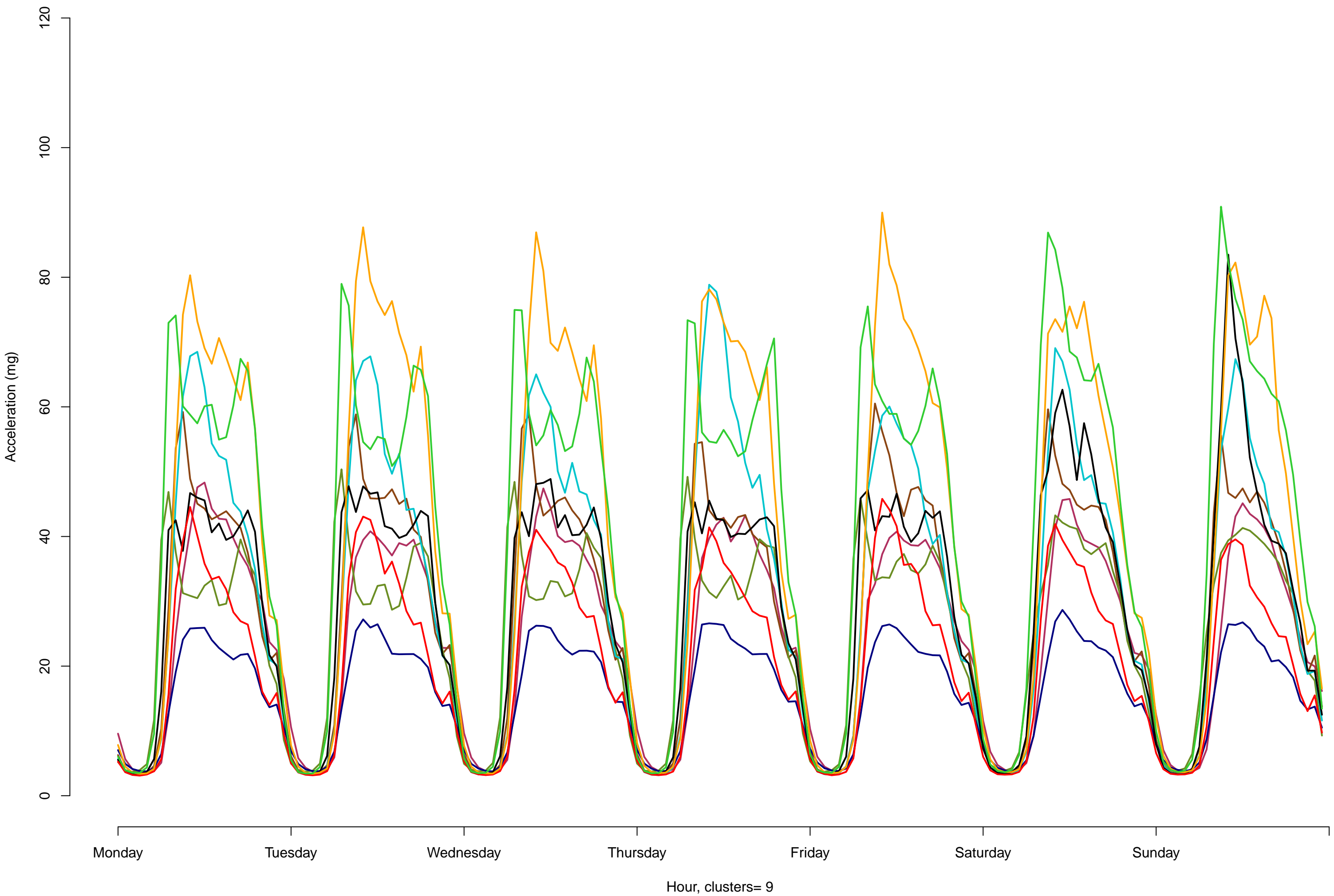

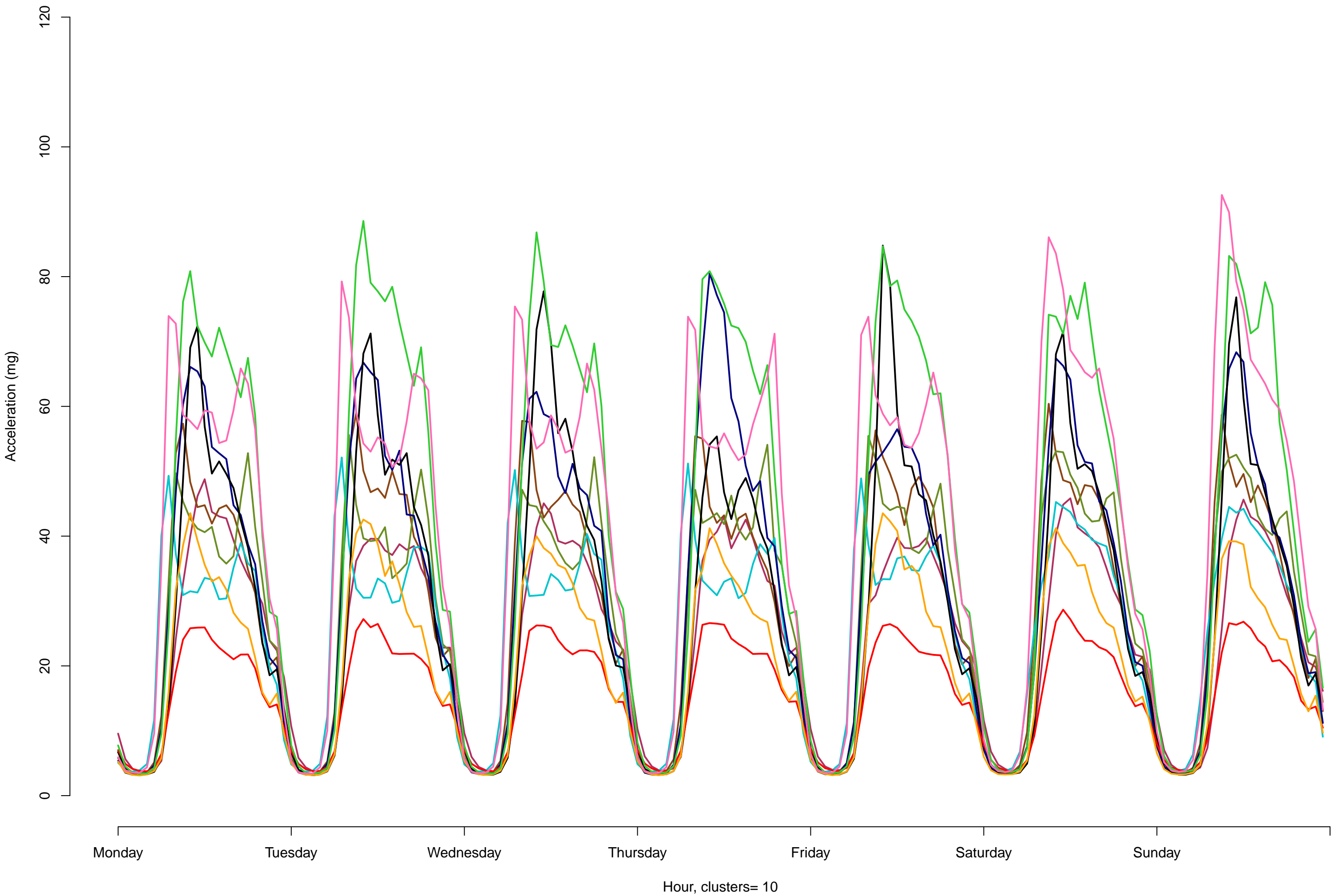

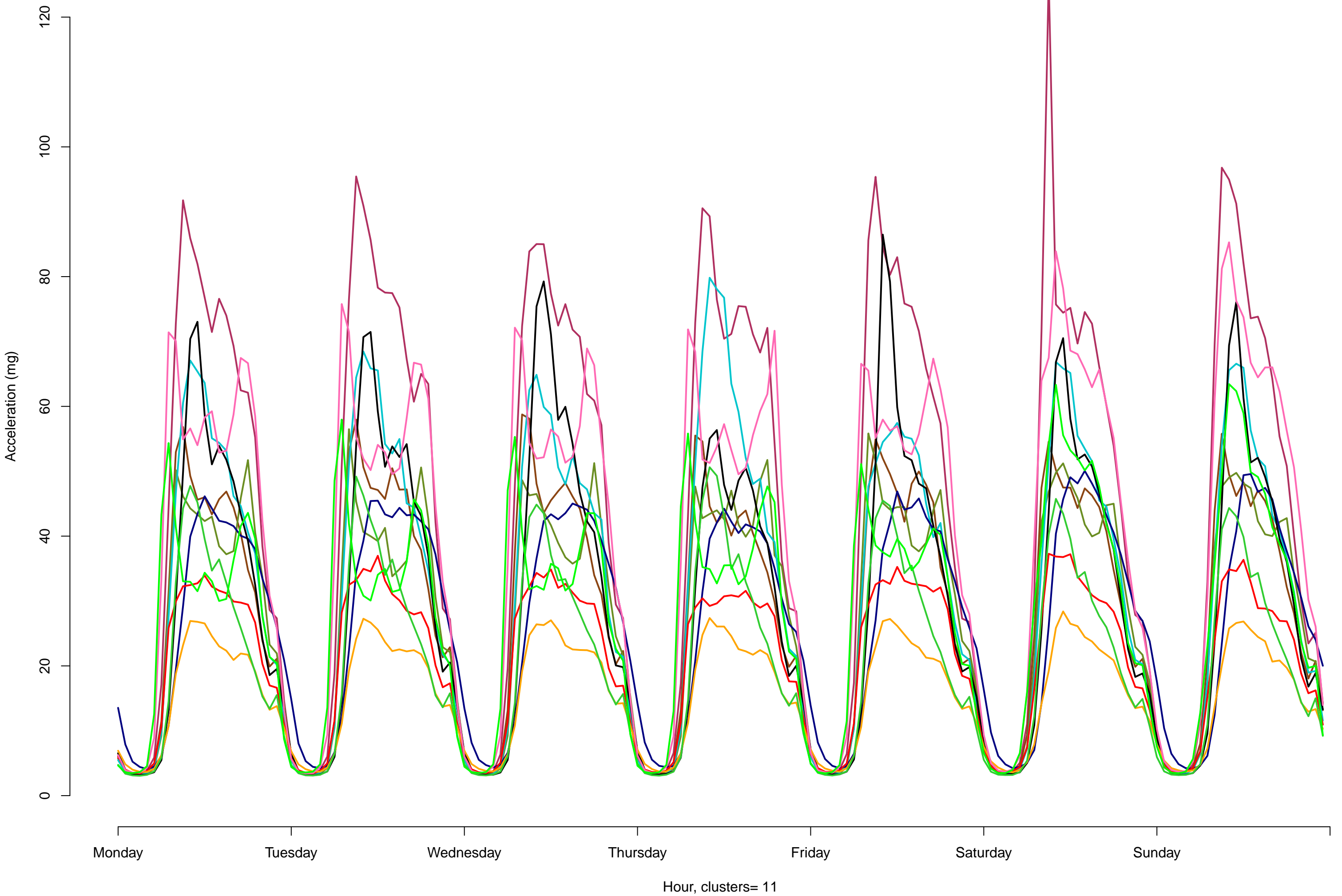

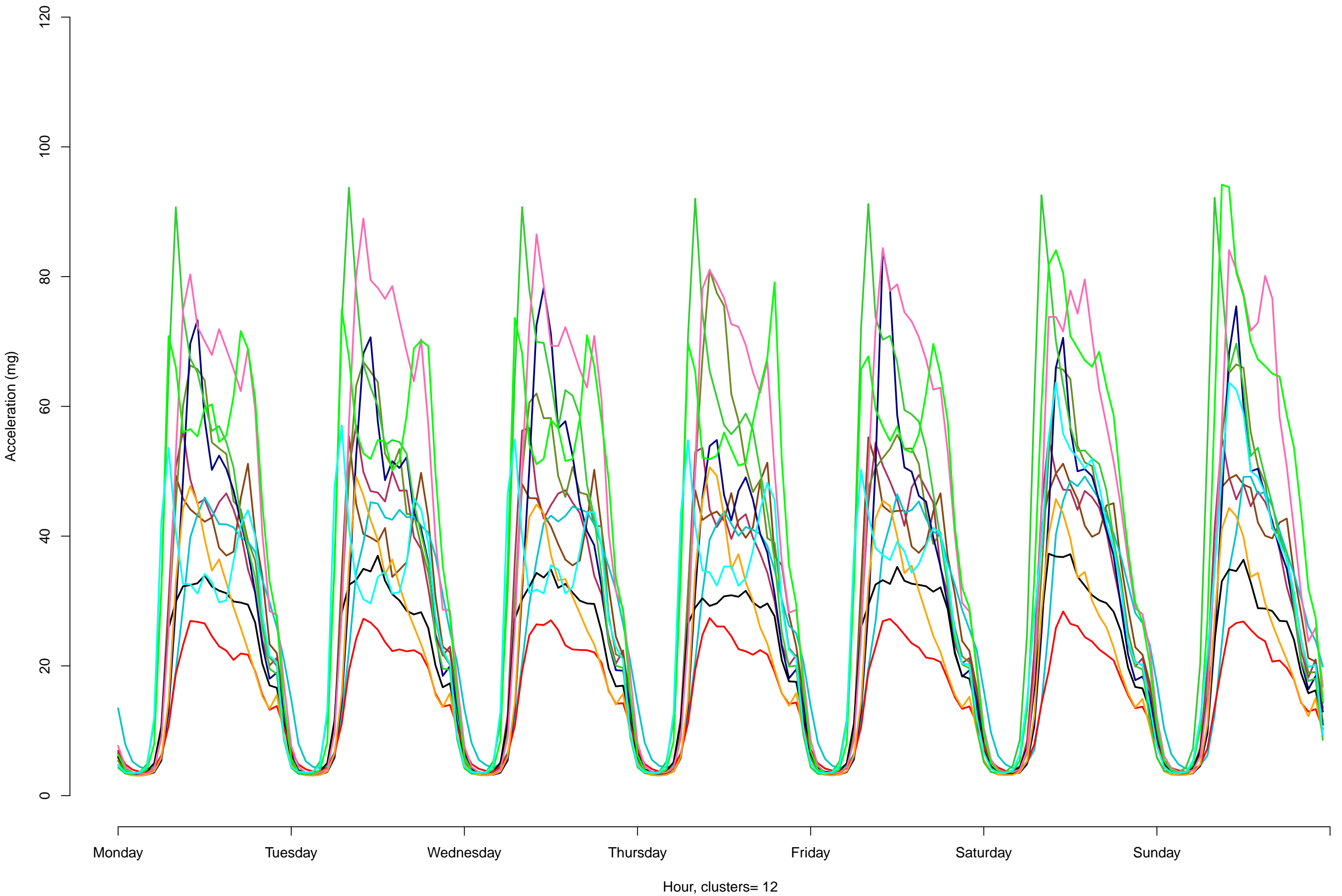

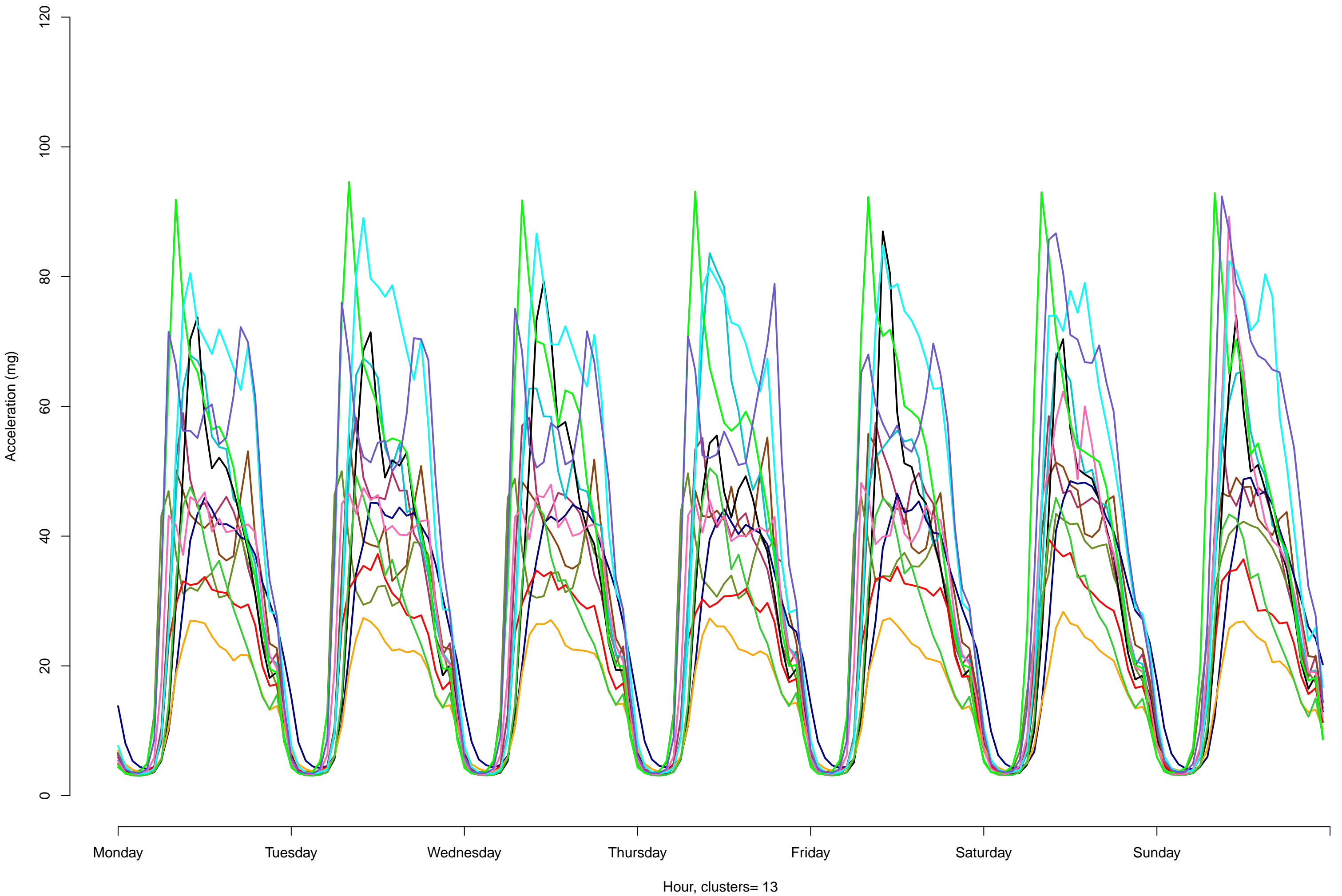

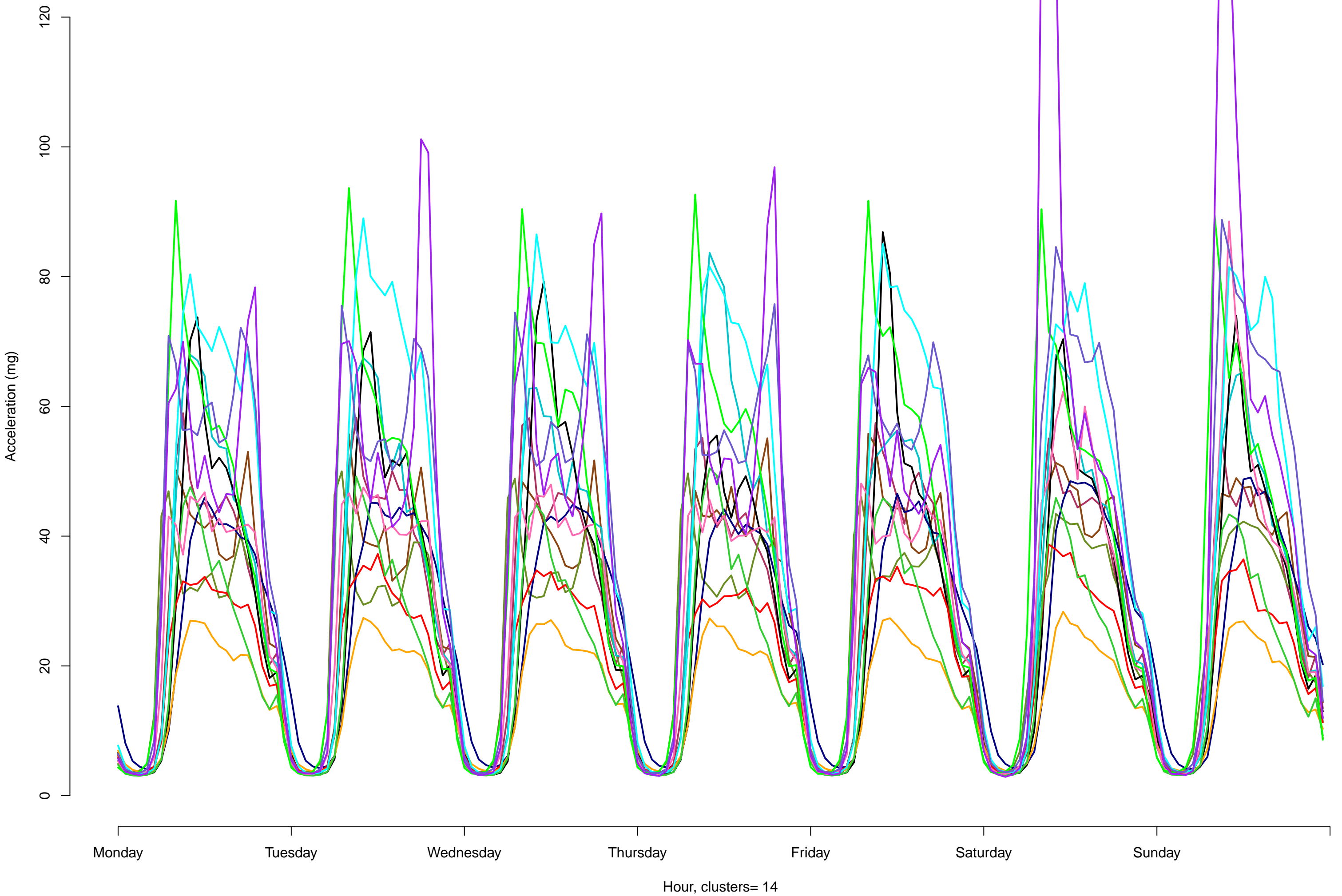

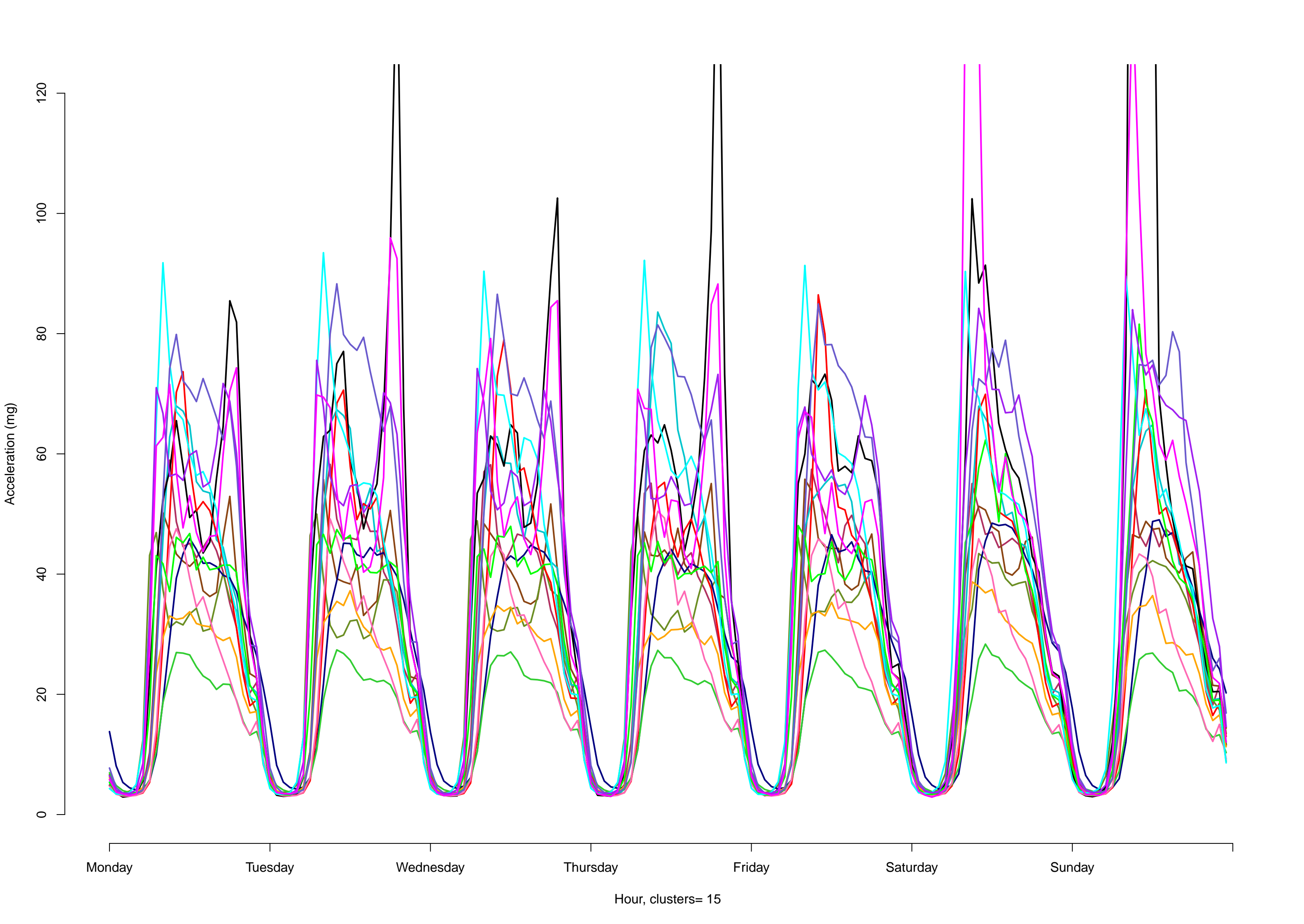

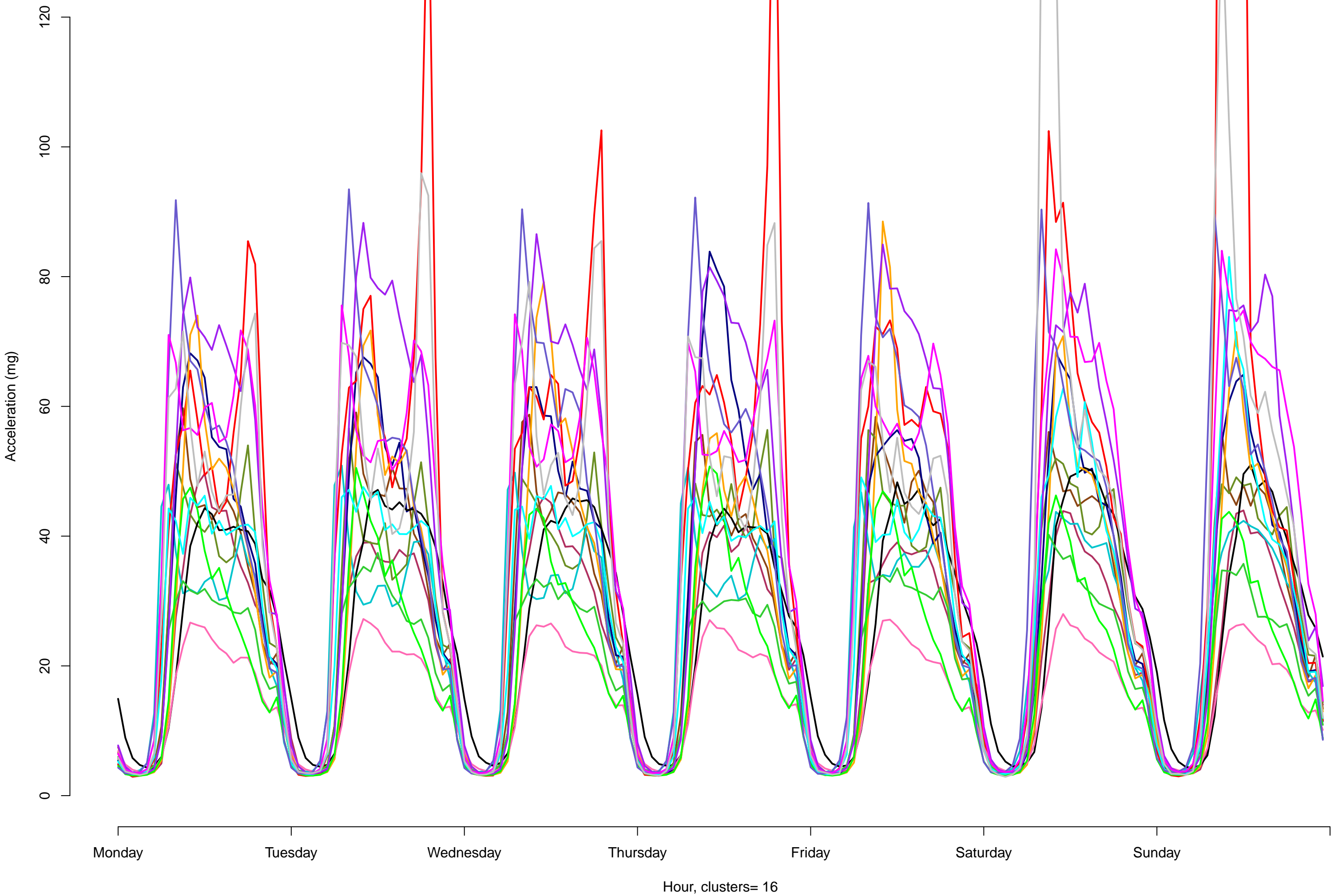

Supplement: Supplementary file 1 [file sensors-21-08220-s001.zip › Supplementaryk_Medoids_average_all.pdf]
